# Organization and post-transcriptional processing of focal adhesion kinase gene

**DOI:** 10.1186/1471-2164-7-198

**Published:** 2006-08-04

**Authors:** Jean-Marc Corsi, Evelyne Rouer, Jean-Antoine Girault, Hervé Enslen

**Affiliations:** 1 Unité Mixte de Recherche-Santé (UMR-S) 536, Institut National de la Santé et de la Recherche Médicale (INSERM) F-75005, Paris, France; Université Pierre et Marie Curie-Paris 6, F-75005, Paris, France; Institut du Fer-à-Moulin, F-75005, Paris, France

## Abstract

**Background:**

Focal adhesion kinase (FAK) is a non-receptor tyrosine kinase critical for processes ranging from embryo development to cancer progression. Although isoforms with specific molecular and functional properties have been characterized in rodents and chicken, the organization of FAK gene throughout phylogeny and its potential to generate multiple isoforms are not well understood. Here, we study the phylogeny of FAK, the organization of its gene, and its post-transcriptional processing in rodents and human.

**Results:**

A single orthologue of FAK and the related PYK2 was found in non-vertebrate species. Gene duplication probably occurred in deuterostomes after the echinoderma embranchment, leading to the evolution of PYK2 with distinct properties. The amino acid sequence of FAK and PYK2 is conserved in their functional domains but not in their linker regions, with the absence of autophosphorylation site in *C. elegans*. Comparison of mouse and human FAK genes revealed the existence of multiple combinations of conserved and non-conserved 5'-untranslated exons in FAK transcripts suggesting a complex regulation of their expression. Four alternatively spliced coding exons (13, 14, 16, and 31), previously described in rodents, are highly conserved in vertebrates. Cis-regulatory elements known to regulate alternative splicing were found in conserved alternative exons of FAK or in the flanking introns. In contrast, other reported human variant exons were restricted to *Homo sapiens*, and, in some cases, other primates. Several of these non-conserved exons may correspond to transposable elements. The inclusion of conserved alternative exons was examined by RT-PCR in mouse and human brain during development. Inclusion of exons 14 and 16 peaked at the end of embryonic life, whereas inclusion of exon 13 increased steadily until adulthood. Study of various tissues showed that inclusion of these exons also occurred, independently from each other, in a tissue-specific fashion.

**Conclusion:**

The alternative coding exons 13, 14, 16, and 31 are highly conserved in vertebrates and their inclusion in mRNA is tightly but independently regulated. These exons may therefore be crucial for FAK function in specific tissues or during development. Conversely pathological disturbance of the expression of FAK and of its isoforms could lead to abnormal cellular regulation.

## Background

Focal adhesion kinase (FAK) is a non-receptor tyrosine kinase highly enriched in focal adhesions [1,2]. FAK is activated following integrins engagement or stimulation of a variety of transmembrane receptors [3]. FAK is closely related (45% amino acid identity) to proline-rich tyrosine kinase 2 (PYK2, [4]), also known as cell adhesion kinase β (CAK β, [5]), related adhesion focal tyrosine kinase (RAFTK, [6]), or calcium dependent protein tyrosine kinase (CADTK, [7]).

FAK comprises three well-defined domains: an N-terminal four-point-one, ezrin, radixin, moesin (FERM domain see [8,9]), a central catalytic domain, and a C-terminal focal adhesion targeting (FAT) domain, necessary and sufficient for the recruitment of FAK to focal adhesions [10]. The structure of these 3 domains has been experimentally determined [11-13]. In mammals or chick the FERM and kinase domains are joined by a short linker (~44 residues) containing a proline-rich motif (PR1), which binds SH3 domains of several proteins including Src and Trio [14-16], and the autophosphorylated Tyr-397 [17-19]. The kinase and FAT domains are joined by a relatively long, poorly characterized, region of ~233 residues containing two proline-rich motifs (PR2, PR3), which provide the basis for many SH3-mediated protein-protein interactions of FAK [3].

FAK is ubiquitously expressed in adult tissues and plays a critical role during embryogenesis as indicated by the lethality of its deletion at 8.5 embryonic days in mice [20]. FAK regulates major cellular functions including migration, spreading, cell cycle progression and survival in numerous cell types [21]. FAK is important in brain development [22,23] and appears to play a critical role in the formation of tumors and in malignancy of cancer cells, controlling their invasive and metastatic capacities (see [24]). PYK2 is functionally distinct as it is involved in signalling pathways initiated by extracellular signals that elevate intracellular calcium concentration and by stressful stimuli (reviews in [25,26]).

FAK is activated by autophosphorylation of Tyr-397 which recruits several SH2 domain containing proteins including Src family kinases (see refs in [25]). Phosphorylation of other tyrosine residues in FAK by these kinases increases its activity and promotes the recruitment and phosphorylation of associated proteins (review in [27]). Thus, FAK acts as an autophosphorylation-regulated scaffolding protein, which triggers the assembly of multimolecular complexes regulating downstream signalling cascades such as the mitogen-activated protein kinase (MAP-kinase) and phosphatidylinositol 3-kinase (PI3-kinase) pathways.

FAK has been highly conserved through evolution. FAK orthologues regulate cell adhesion and migration in drosophila [28-32] and sea urchin [33] and play an essential role in the morphogenesis of zebrafish [34,35]. In some species, multiple FAK transcripts resulting from alternative splicing and/or promoter usage have been characterized. An alternative internal promoter has been identified in chicken and mouse [36,37]. Transcription from this alternative promoter results in the production of a truncated isoform of FAK, lacking its N-terminal and catalytic domains, termed FRNK (FAK-related non-kinase) [36]. FRNK acts as a dominant negative, inhibiting numerous effects of FAK [21]. Interestingly an N-terminal-truncated form of PYK2, named PRNK (PYK2-related non kinase), has also been reported [38].

Cloning FAK transcripts from rat brain revealed the existence of various 5'-leader sequences, and alternative splice variants predicting changes in the amino acid sequence of FAK [39,40]. These alternative exons code for small peptides located either just before the FAT domain (3 residues: Pro-Trp-Arg defining the FAK^+ ^isoform), or on either side of the autophosphorylated Tyr-397 (boxes 28, 6 and 7, in reference to the number of amino acids encoded by these exons). FAK isoforms including boxes 6 and 7 (FAK^6,7^) have a much higher autophosphorylation on Tyr-397 than the "standard" isoform (FAK^0^), which does not include them [40-42]. FAK^+6,7 ^is the predominant isoform in rat and mouse brain [40,41]. In addition, a study in human reported the possible existence of other mRNA variants of FAK, which could potentially lead to the translation of truncated FAK proteins [43]. The expression of multiple isoforms of FAK provides a potential for multiple regulations and/or functions in specific cell types and/or in pathological conditions including cancer.

The aim of the present study was to take advantage of the available genomic sequence information from multiple species to better define the organization of FAK gene. Using sequence analysis and RT-PCR we then examined the existence and conservation of various putative variants of FAK transcripts. Finally, we investigated thoroughly in rodent and human tissues the expression pattern of conserved alternative splicing likely to be biologically significant.

## Results

### Phylogeny of FAK family kinases

**Figure 1:**
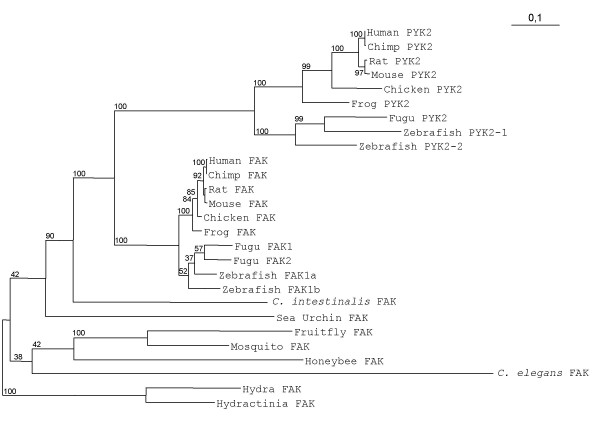
**Phylogenetic analysis of FAK and PYK2**. Blocks of conserved positions distributed across the full length FAK sequences from 16 species were used in a maximum likelihood analysis. The putative FAK sequences from cnidarians (hydra and hydractinia) were set as the outgroup. The branch lengths of the tree are proportional to differences between species. Numbers beside branch points indicate the confidence level for the relationship of the paired sequences as determined by bootstrap statistical analysis (100 replicates). See text for a discussion of FAK duplication in fugu and zebrafish.

FAK cDNA and/or genomic sequences are known in several vertebrate and non-vertebrate species. PYK2, which shares around 45% amino acid identity with FAK, has been identified in mammals [4-7]. FAK gene is referred to as *Ptk2 *or *Ptk2a*, and PYK2 gene as *Ptk2b*. *Ptk2a *and *Ptk2b *are both located on chromosome 8 in human (locus 8q24-qter and 8p21.1 respectively) and chimpanzee, but these two genes are not syntenic in other mammalian species. A recent phylogenetic study of FAK family suggested that its common ancestor with PYK2 was very ancient [33]. We took advantage of the availability of sequence information in an increasing number of species, to re-investigate the overall phylogenetic relationships between FAK family members and PYK2. We aligned FAK and PYK2 sequences from 16 species (see additional file 1) and used a computerized method (Gblocks) that eliminates poorly aligned positions and divergent regions that may not be homologous or may have been saturated by multiple substitutions [44]. Thus, our analysis was performed on blocks of positions distributed across the full length FAK and PYK2 sequences. The phylogenic tree resulting from a maximum likelihood analysis based on these blocks of sequence is shown in Fig. 1. The tree suggests that gene duplication leading to the appearance of FAK and PYK2 occurred after the urochordate (*Ciona intestinalis*) branch since distinct clusters of FAK and PYK2 proteins are only observed in vertebrates. Furthermore, the vertebrate FAK genes are more closely related to the common ancestor than PYK2 genes, suggesting that PYK2 was subjected to less evolutionary pressure. The global tree topology obtained with the maximum likelihood analysis was reproduced using the distance method analysis (see Methods section) on FAK complete amino acid sequence (with or without Gblocks sequence alignment processing) and on isolated FERM or kinase domains (data not shown). Interestingly, the zebrafish genome includes two distinct and likely functional FAK genes [34,35] presumably resulting from genome duplication [45]. Similarly two FAK genes are predicted in fugu genome (Ensembl V38). In most analysis fugu FAK proteins formed a distinct monophyletic group supporting independent duplication in each fish species (Fig. 1). However, maximun likelihood analysis with FERM domain indicated fugu FAK2 and zebrafish FAK1a as monophyletic, suggesting that these proteins are orthologues and that FAK gene was duplicated before emergence of these two fish species, in agreement with the usual view of early genome duplication in this clade [45]. Altogether, these data suggest that vertebrate FAK family members evolved from a common ancestral gene, which was duplicated in deuterostomes after the echinoderma embranchment, leading to the evolution of PYK2 with distinct properties. Thus, FAK and PYK2 appear to be coded by paralogous genes in vertebrates.

**Figure 2:**
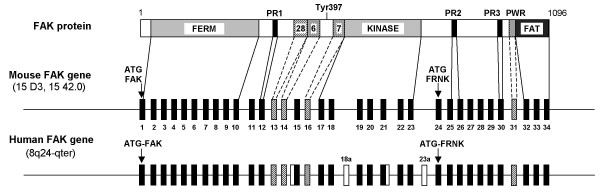
**Comparison of the organization of coding regions of murine and human FAK genes**. The correspondance is shown between the domain organization of FAK protein and the structure of the murine and human FAK genes. Dark and striped boxes denote constitutive and conserved alternative exons, respectively. White boxes denote species-specific exons or additional sequences reported in transcripts (see Table 1 for details). Translation initiation codon (**ATG**) for FAK and FRNK are indicated by arrows. Exons 18a and 23a are specific of primate genomes. FERM: four-point-one, ezrin, radixin, moesin; PR: proline-rich; FAT: focal adhesion targeting. The positions of autophosphorylated Tyr-397, boxes 28, 6, 7 and PWR are indicated.

FAK is conserved in vertebrate and non-vertebrate species (see additional file 1). The most conserved domain is the kinase domain (lowest sequence identity: e.g. human vs *C. elegans*: 50 %) followed by the FERM domain (lowest sequence identity: e.g. human vs *C. elegans *= 24 %). The *C. elegans *orthologue of FAK appears to be the most divergent. The human FAT domain has only 18% amino acid identity with the C-terminus of the *C. elegans *sequence. However, hydrophobic cluster analysis [46] revealed that the nematode FAK C-terminal region has an organization similar to that of human FAT (data not shown), suggesting that it corresponds to a *bona fide *FAT domain. The sequence of the linker regions between FERM and kinase domains, and between kinase and FAT domains (Fig. 2 and see additional file 1) is less conserved. The FERM-kinase linker contains a conserved sequence in FAK and PYK2, (S/T)(D/E)DYAEI, with a tyrosine that has been shown to be autophosphorylated in mammals and drosophila. This sequence is absent, however, from the available genomic sequence of *C. elegans *FAK orthologue (see additional file 1), suggesting that FAK has a fundamental autophosphorylation-independent biological function. The linker region between kinase and FAT domains is the most variable region, and contains insertions in some species, which are longest in echinoderma (sea urchin), cniderias (hydra and hydractinia) and arthropods (drosophila, mosquito and honeybee). Interestingly the *C. elegans *predicted sequence does not encompass proline-rich motifs in this region (additional file 1).

### Organization of murine and human FAK genes

We then focused our study on mammalian FAK, and to obtain more insights into the organization of its gene we compared in detail its sequence in human and rodents.

#### FAK and FRNK promoters

FAK promoter has been characterized in human [47], while the internal FRNK promoter has been identified in chicken and mouse [36,37]. We identified the orthologous mouse FAK and human FRNK promoters (see additional files 2, 3 and 4). Pairwise conservation analysis of these sequences showed 2 evolutionary conserved regions (ECR, more than 70% identity) in FAK promoter region (additional file 3). In FRNK promoter, we identified a novel ECR (ECR3, additional file 4) located upstream from the previously reported ones [48]. We searched FAK and FRNK promoters for conserved transcription factors binding sites, and identified novel putative binding sites (see additional files 3 and 4), in addition to those previously reported [47,48].

#### FAK 5'-untranslated region

**Figure 3:**
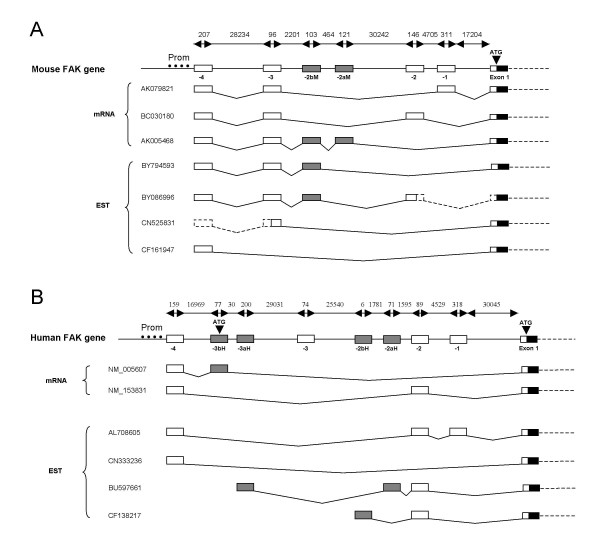
**Genomic structure and alternative splicing of the 5' regions of murine and human FAK genes**. Genomic structure of the 5'-untranslated region of mouse (**A**) and human (**B**) FAK genes with reported transcripts and ESTs from each species. The lengths (bp) of DNA fragments are shown on top of each genomic gene structure. The characterized human promoter [47] is symbolized with black dots (Prom) in **B**, and the conserved regions located on the mouse gene are indicated (Prom) in **A**. Exons -1, -2, -3, and -4 are conserved between mouse and human (87% 60%, 72% and 74% identity respectively) and indicated by open boxes. Species specific exons are indicated by grey boxes. Human exons -4, -3bH, and -2 as well as all murine exons were already annotated in NCBI gene Entrez and Ensembl databases. The accession number of each transcript is reported. Dashed lines indicate putative splicing of mouse transcripts with incomplete 5' UTR regions (ESTs [DDBJ:BY086996, GenBank: CN525831]

We next investigated the genomic organization of the 5'-untranslated region. This region is important since in most cases the rate limiting step of translation is initiation, which implicates the 5'-untranslated region (UTR) [49]. FAK transcripts cloned from rat brain revealed several 5'-leader sequences containing various combinations of five sequences termed boxes A-E [39] (sequence E was later shown likely to be a cloning artifact, Studler and Girault unpublished observations). We aligned the murine and human 5' UTR sequences of the FAK transcripts reported in either mRNA or EST databases (Fig. 3A and 3B) and localized them in the murine and human FAK gene (see additional file 2). All reported transcripts contain box A, which is the untranslated 5' part of the first coding exon. Murine transcripts contain various combinations of 5' UTR sequences corresponding to six previously annotated exons (NCBI and Ensembl databases) (Fig. 3A). Comparison of human sequences revealed the existence of at least eight exons, three of which have been annotated (Fig. 3B). We numbered the four exons conserved between human and mouse as exons -1, -2, -3 and -4 (in order of increasing distance from the translation initiation site). Boxes B, C and D, previously characterized in rat, correspond to exons -1, -3 and -4 respectively (Fig. 3). Exon -3 is present in the human gene, but has not yet been reported in any transcript in that species (Fig. 3). The two other mouse 5'-untranslated exons are not conserved in human and we annotated them as -2aM and -2bM. Conversely, four human untranslated exons are not conserved in mouse FAK gene and we numbered them as exons -2aH, -2bH, -3aH, and -3bH (Fig. 3B). The homology of exons -2aM, -3aH and -3bH with various SINE/ALU repeated sequences (data not shown) suggests they are part of mobile elements, an observation which may account for their lack of evolutionary conservation. Altogether these results identify five novel putative 5'-untranslated exons (-3aH, -3, -2bH, -2aH and -1) upstream from the canonical initiation codon of the human FAK gene. Our results suggest a complex regulation of exons inclusion/exclusion at the 5' end of FAK mRNA, which may be important in their localization, stability and/or expression. All the mouse transcripts detailed in Fig. 3A contain the conserved exon -4. It is noteworthy that all the human and mouse transcripts detailed in Fig. 3A and 3B are compatible with the existence of a single promoter region (see above) adjacent to exon -4 in both species (Fig. 3) as proposed by Golubovskaya et al [47] for human FAK.

#### FAK coding sequence

Concerning the coding sequence, 34 exons are annotated in human (NCBI Gene ID: 5747) (see additional file 5) and rat (NCBI Gene ID: 25614) FAK genes, including those coding for boxes 28, 6, 7 and Pro-Trp-Arg (PWR, characterizing FAK^+^). In contrast, the exon encoding PWR has not been annotated in the mouse gene (NCBI Gene ID: 14083), although isoforms containing this peptide have been characterized in mouse [39]. We examined the sequence of the mouse FAK gene and identified an exon encoding PWR. We therefore propose a consistent annotation of human and mouse FAK genes, and number exons encoding boxes 28, 6, 7 and PWR as exons 13, 14, 16 and 31 respectively, in both genes (Fig. 2 and see additional files 2 and 6).

### Conserved alternative splicing of FAK coding sequence

**Figure 4:**
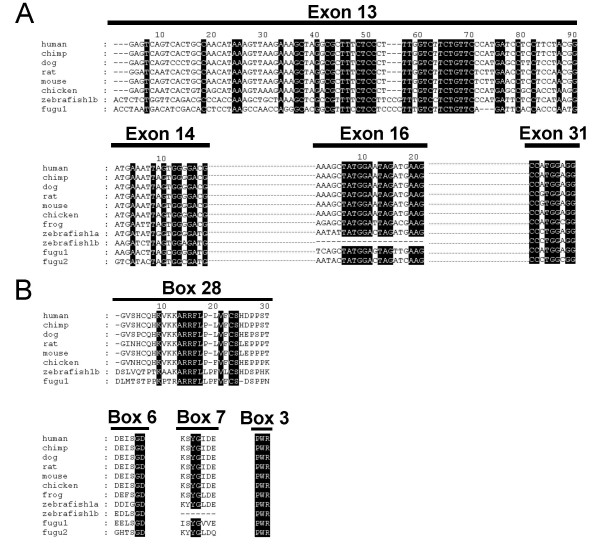
**Conservation of exons 13, 14, 16 and PWR during evolution**. (**A**) Alignment of exons 13, 14, 16 and 31 nucleotides sequences of human, chimp, dog, rat, mouse, chicken, zebrafish and fugu FAK genes. Alignment gaps are indicated by dashes. Sequences identical in all species are highlighted in dark. Fugu and zebrafish genomes contain two FAK genes (FAK1 and FAK2, and FAK1a and FAK1b, respectively). Exon 13 was found in only one zebrafish and fugu FAK gene, whereas exon 16 was found in only one zebrafish FAK gene. (**B**) Alignment of the amino acid sequences of boxes 28, 6, 7 and 3 (PWR) of the same species as in **A**. Alignment gaps are indicated by dashes. Amino acids identical in all species are highlighted in dark.

Several alternative exons coding for short peptides have been reported in rat and mouse FAK [39,40]. Exons 13, 14, 16 and 31 are conserved in mouse, rat and human, and their presence, except for exon 13, has also been reported in transcripts from frog [50,51]. We examined the presence of these exons in the FAK genes of *Homo sapiens*, chimpanzee (*Pan troglodytes*), dog (*Canis familiaris*), chicken (*Gallus gallus*), frog (*Xenopus laevis and Xenopus tropicalis*), fugu (*Takifugu rubripes*), and zebrafish (*Danio rerio*). We identified exons 14, 16 and 31 in the FAK genomic sequences of chimpanzee, dog, chicken, frog, fugu, and zebrafish, with more than 80% identity in their nucleotide sequences (Fig. 4A). This corresponds to 60–80% amino acids identity for boxes 6 and 7 and 100% for PWR (Fig. 4B). Interestingly, exon 16 was found in only one FAK zebrafish gene (zebrafish 1a). Exon 13 (encoding box 28) was found in the genomic sequences from dog and chicken and in only one FAK gene from zebrafish (zebrafish 1b) and fugu (fugu1) (80% identity for nucleotides and amino acids sequences) (Fig. 4A and 4B). We could not conclude about the presence of exon 13 in frog since FAK gene sequence is incomplete in this species. None of these alternative exons could be identified in non-vertebrate species, suggesting that alternative exons 13, 14, 16 and 31 appeared in a common ancestor of vertebrates. The high degree of conservation of nucleotide and amino acid sequences supports an important physiological role of FAK isoforms containing these alternative spliced exons.

### Putative additional variant FAK transcripts

As mentioned above two products, FAK and FRNK, are transcribed and translated from FAK gene and more variety is generated by alternative splicing of four highly conserved exons. In addition to these well characterized gene products, a number of cDNAs and ESTs have been reported that include additional variations. In particular, several human FAK transcripts containing various deletions and/or insertions have been reported in different tissues (Table 1). Some of these transcripts would encode putative proteins with interesting predicted properties. Alternatively, these variant transcripts could correspond to regulatory mechanisms at the mRNA level including nonsense-mediated decay (NMD) [52]. We first examined which of the reported variant FAK ESTs or mRNAs were compatible with the human FAK gene sequence. This ruled out reported transcripts containing a 39-bp sequence, with an ATG in frame with FAK open reading frame (ORF) apparently inserted in the middle of exon 5 [43]. This sequence was found in chromosome 7, and not in FAK gene, located on chromosome 8, suggesting that it may have resulted from chromosomal abnormality in the source material.

We then analyzed human adult brain RNA by RT-PCR to search for the presence of FAK transcripts containing other reported modifications compatible with the genomic sequence (Table 1). One transcript identified in stomach [Refseq:NM_005607] included an additional 77 nt sequence 5' of the canonical exon 1 of FAK (Table 1). This transcript corresponds to the splicing of exon -3bH (Fig. 3) with exon 1. Although exon -3bH is also found in the chimpanzee FAK gene, it has not been reported in any transcript in that species. Interestingly, exon -3bH contains an ATG in frame with FAK open reading frame (ORF), which would give rise to a 25-amino acid N-terminal elongation of FAK [Refseq:NP_005598] (Table 1). However, this ATG diverges from the canonical consensus sequence [53] and its use remains to be demonstrated. PCR reactions using a forward primer spanning the boundary between exons -3bH and 1 (primer H-F(-3bH)) and a reverse primer spanning the boundary between exons 5 and 6 (H-R1) did not yield *bona fide *amplification products, suggesting that this exon is not included in adult human brain FAK transcripts (data not shown).

A FAK transcript truncated of the first 14 exons and containing an additional 135 nucleotides sequence 5' of exon 15 has been isolated from human hippocampus mRNA [GenBank: BC028733]. This additional sequence was also reported in several macaque transcripts (Table 1) and we identified it in intron 14, adjacent to exon 15 in the human FAK gene (Table 1). This transcript contains a non-canonical ATG (60 nucleotides upstream from exon 15) in frame with FAK ORF. RT-PCR with a forward primer located in this sequence (H-F7) and reverse primers located in exon 18 (H-R3) amplified a fragment of the expected size (data not shown). Its sequencing confirmed the presence of exons 15, 17, and 18, and the absence of exon 16 (data not shown). No PCR products were obtained with forward primers located in various exons upstream from exon 15 and a reverse primer located in the additional nucleotide sequence (data not shown). Thus, this transcript could correspond to an alternative splice variant, or be generated from an alternative promoter located in intron 14. At any rate, our results demonstrate that this transcript is expressed in adult human brain. If translated, it would encode a FAK isoform deleted of the FERM domain, but possibly containing a 20-residue N-terminal extension [GenBank: AAH28733] (Table 1).

FAK transcripts containing an in-frame insertion of 85 nucleotides in the catalytic domain have been reported in human brain [43]. As expected we localized this sequence between exons 18 and 19 in the human FAK gene and annotated it as exon 18a (Fig. 2, Table 1 and see additional files 2 and 6). Exon 18a is flanked by canonical splice donor and acceptor sites. This exon is conserved in *Homo sapiens *and *Pan troglodytes *genomes, although in both species two nucleotides are missing as compared to the published human cDNA sequence, predicting a frame shift and the insertion of a stop codon in exon 19 (Table 1). Sequencing of PCR products (primers H-F9/H-R4) obtained from adult human brain cDNAs confirmed the existence of exon 18a, the nucleotides deletion and the frame shift (data not shown).

A 3' truncated FAK transcript cloned from human brain and characterized by a 91-nucleotide insertion was also reported [43]. We localized this additional sequence, which contains an in-frame stop codon, between exons 23 and 24, and we annotated it as exon 23a (Fig. 2, Table 1 and see additional files 2 and 6). The presence of this exon and its sequence were confirmed by PCR (primers H-F11/H-R6) of human brain cDNAs (data not shown). Transcripts containing either 18A or 23A would encode C-terminally truncated isoforms of FAK (respectively [GenBank: L05186] [Swiss-Prot: Q05397-(2, 3, 4)] and [Swiss-Prot: Q05397-3], see also Table 1). We found exons 18a and 23a in *Pan troglodytes*, but not in *Mus musculus *or *Rattus norvegicus *FAK gene. No transcript containing these sequences has yet been reported in chimpanzee. The analysis of repeated sequences in these regions revealed the existence of putative long interspersed elements (LINEs), an abundant class of retrotransposons very active in the human genome [54]. Altogether our analyses indicate that these two variant mRNAs are present in human tissues and would lead to a truncated protein. However, their lack of conservation suggests that they may not be biologically important and may reflect the unstability of the corresponding DNA regions.

A FAK transcript (containing exon 18a) in which intron 21 is not spliced at its canonical 5' border, resulting in an additional 15 nt and a stop codon, has also been reported in human brain [Swiss-Prot: Q05397-4] [43]. We found this sequence at its expected localization in intron 21 of FAK (Fig. 2). However, the human genome sequence reveals 1 nucleotide substitution in the 15 bp additional sequence compared to the published sequence resulting in a C-terminal glycine instead of a glutamic acid (Table 1). Using a forward primer located in exon 18 (H-F8) and a reverse primer (H-R5) overlapping the end of exon 21 and the additional 15 nt sequence, we obtained a PCR product of the expected size, albeit devoid of exon 18a (data not shown and Table 1). This transcript, similarly to those containing exon 18a, would encode a FAK isoform deleted of the C-terminal moiety of the protein including most of the kinase domain.

Various FAK transcripts appear to be lacking specific exons. A transcript missing exon 26 has been reported in human placenta [GenBank: BC035404] and dog [RefSeq:XM 851079.1] (Table 1). Our data indicate that if this variant is expressed in humain brain this occurs at a very low level (primers H-F10/H-R7). In contrast, we clearly confirmed by RT-PCR (data not shown), the expression of another FAK variant cloned from brain [GenBank: L05186] in which exon 28 is skipped (primers H-F12/H-R8) (Table 1). No change in ORF results from the absence of exon 28. During this study, we also found 2 other FAK transcripts lacking either exon 2 or exon 29 (primers H-F1/H-R1 and H-F12/H-R9 respectively) (Table 1). The skipping of these exons leads to stop codons in exons 3 and 32, respectively (Table 1). It is noteworthy that deletion of exon 2 was used to generate endothelial cell-specific FAK knockout mice [55].

Altogether these results demonstrate that multiple FAK transcripts are expressed in adult human brain (Table 1). However, these transcripts appear to be expressed at low levels in *Homo sapiens *and not to be conserved in other species but in some cases *Pan troglodytes *or *Macaca fascicularis*. These observations suggest that these alternative transcripts may be side products and/or play a regulatory role in transcription.

### Regulatory elements possibly involved in the control of FAK alternative splicing

**Figure 5:**
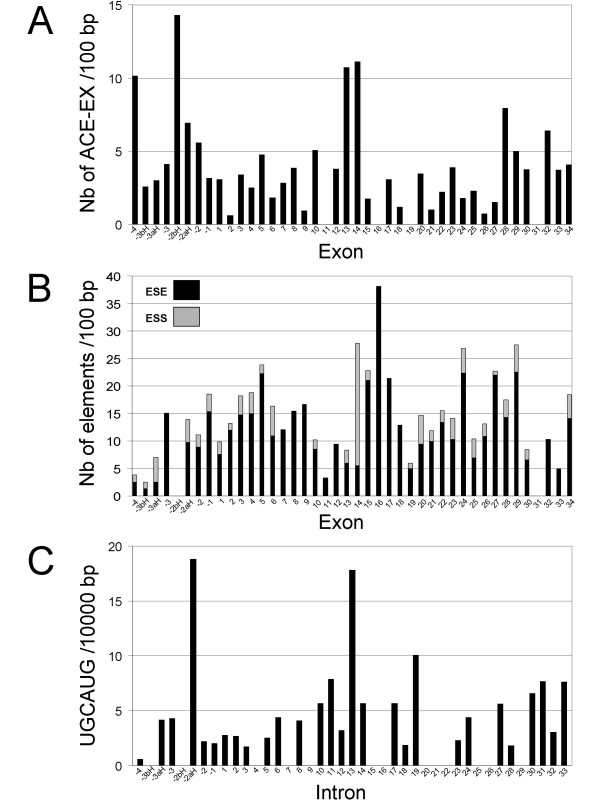
**Cis-regulatory elements of alternative splicing in the human FAK gene**. Known cis-regulatory elements involved in mRNA alternative splicing were searched in the human FAK gene (NCBI Gene ID: 5747) as described in the Methods section. (**A**) Frequency of ACE-EXs per 100 bp in each FAK exon. (**B**) Frequencies of exonic splicing enhancers (ESE, black) and exonic splicing silencers (ESS, grey) per 100 bp in each FAK exon (for example 5 ESE and 23 ESS/100 bp were found in exon 14). (**C**) Frequency of the hexamer UGCAUG per 10,000 bp in each FAK intron (numbered according to their 5' flanking exon).

Since FAK gene products appear to undergo multiple alternative splicing, we searched for genomic elements which might be involved in this process. Alternative inclusion/exclusion of exons is known to be regulated by cis-regulatory elements present in the exon and within the flanking introns. Using the web-based program Acescan2, we searched for sequences referred to as ACE-EXs which are overrepresented in alternative evolutionary conserved exons [56]. We observed a marked enrichment of ACE-EXs sequences in conserved alternatively spliced exons -4, 13 and 14 (Fig. 5A). Other cis-regulatory sequences are exonic and intronic splicing enhancers (ESE and ISE, respectively) or silencers (ESS and ISS, respectively) that control exon skipping by recruiting trans-acting splicing factors [57,58]. We analyzed the human FAK gene with the RESCUE-ESE prediction program [59] to determine the frequency of ESE per exon and found a high score for exon 16 (Fig. 5B). The same results were obtained by browsing the murine FAK gene (data not shown) in which exon 16 inclusion/exclusion is also alternatively regulated. The search for ESS with two different programs (Acescan2 and the EBI ASD tools) [60-62] indicated the presence of a high number of ESS in exon 14 in human and mouse FAK gene (Fig. 5B and data not shown). Interestingly there was no enrichment of ESE or ESS in the 5' non coding exons (Fig. 5B). On the other hand, we found a high occurrence of the hexamer UGCAUG, a well-characterized ISE in various genes [63], in the intronic regions between the alternatively spliced exons -2aH and -2 (intron -2aH) and between exons 13 and 14 (intron 13) of human FAK gene (Fig. 5C). These results provide a basis for the possible regulation of alternative inclusion/exclusion of exons located in the 5' UTR region or in the canonical coding region of human and mouse FAK gene.

### Developmental regulation of alternative splicing of FAK exons 13, 14, and 16 in mouse brain

**Figure 6:**
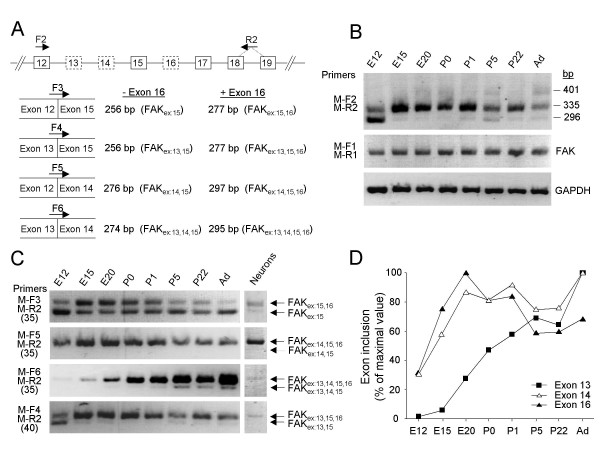
**Alternative splicing of FAK exons 13, 14 and 16 in mouse brain during development**. (**A**) RT-PCR strategy. Top: Genomic organization of the FAK gene (exons 12–19); alternatively spliced exons are boxed with dotted lines. The positions of the primers are indicated. PCR with the flanking forward (F2) and reverse (R2) primers amplified simultaneously all exon combinations. The selective targeting of FAK alternative transcripts was achieved with specific forward primers (F3, F4, F5, F6) coupled to the R2 primer. Each forward primer targeted transcripts with only one combination of exons 12, 13, 14 and 15; the size of the PCR product(s) distinguishes the absence or presence of exon 16 in each transcript. For each species similar primers were used (termed according to the species and the position, see additional file 8), taking into account sequence variations. (**B**) RT-PCR analysis of alternative splicing of exons 13, 14 and 16 during mouse brain development using flanking primers. The position of molecular markers 296 bp, 335 bp and 401 bp correspond to the size of the expected PCR products for FAK_ex:15_, FAK_ex:14,15,16 _and FAK_ex:13,15,16 _respectively. The total levels of FAK transcripts were estimated by RT-PCR with primers (M-F1 and M-R1) located in constitutive exons. Amplification of GAPDH transcripts was used as a control. (**C**) RT-PCR monitoring the developmental expression of alternatively spliced FAK transcripts (left panel) and their expression in E16 cortical neurons (right panel) are shown. The number of PCR cycles used is indicated between parenthesis. (**D**) Pattern of inclusion of FAK alternative exons 13, 14 and 16 during development. Quantification of PCR products was performed as described in Methods. The inclusion of exons 13, 14 or 16 was estimated at each developmental stage by adding the normalized values of all the transcripts containing the corresponding exon and expressed as percent of the maximum.

Our study of FAK phylogeny supports the conservation in vertebrates of alternative splicing of exons 13, 14 and 16. Although this alternative splicing can generate theoretically 8 distinct transcripts, it is unclear which of these transcripts are actually expressed since the combination of the various exons has not been studied systematically. To amplify simultaneously all exons combinations, we used RT-PCR with primers flanking this region of alternative splicing (F2 in exon 12 and R2 spanning exons 18–19, Fig. 6A). Embryonic mouse brain mRNA at different developmental stages was first analyzed (primers M-F2 and M-R2, Fig. 6B). We obtained multiple PCR products migrating on agarose gels as expected for fragments containing various combinations of exons 13, 14 and 16 (Fig. 6B). In the same RNA samples the total levels of FAK transcripts were estimated by amplification of a constitutive fragment, using primers M-F1 and M-R1 located in exons 7 and 9 respectively. Although the total levels of FAK appeared stable throughout development, dramatic alterations were observed in the ratios of the various isoforms. At E12, the short PCR product, with a size indicating it included only exon 15, was predominant. The amplification product of ~340 bp (presumably including exons 14, 15 and 16), increased dramatically at E15 and remained the major isoform until adulthood (Fig. 6B). Longer isoforms, presumably including exon 13, were less abundant and detected only in post-natal brain.

The use of flanking primers has the advantage to allow the comparison of the ratios of the various isoforms between different samples. However, it does not identify accurately the exact combination of the various alternatively spliced exons in FAK transcripts. To address this question we set up an RT-PCR approach using 4 distinct forward primers (F3, F4, F5, F6) spanning each possible exonic boundaries immediately upstream from exon 15 and the R2 reverse primer described above (Fig. 6A). PCR reactions with each forward primer amplified specifically isoforms containing one of the possible combinations of exons 13 and 14. For each of them, the presence of exon 16 generated a distinct longer PCR product (Fig. 6A). We analysed embryonic mouse brain mRNA at different developmental stages using this approach. Except for transcripts with alternative exon 14 alone (FAK_ex:14,15_), which were not detected at any stage (Fig. 6C, M-F5/M-R2), our results suggest that the expression of FAK isoforms containing the various combinations of exons 13, 14 and 16 follows three distinct general patterns during mouse brain development (see additional file 7): i) Transcripts containing no alternative exon (FAK_ex:15_) or containing exon 13 alone (FAK_ex:13,15_) were detected at E12 and decreased during development (Fig. 6C, M-F3/M-R2 and M-F4/M-R2); ii) Transcripts with alternative exon 16 alone (FAK_ex:15,16_), or in combination with 13 or 14 (FAK_ex:13,15,16 _and FAK_ex:14,15,16_), were present at E12, peaked at E20 and tended to decrease thereafter (Fig. 6C, M-F3/M-R2, M-F5/M-R2 and M-F4/M-R2); iii) FAK isoform combining the three alternative exons, FAK_ex:13,14,15,16_, was detectable at E12 and increased steadily thereafter (Fig. 6C, M-F6/M-R2) while transcripts with associated exons 13 and 14 alone (FAK_ex:13,14,15_) followed a similar trend, at very lower levels (Fig. 6C, M-F6/M-R2). It should be noted that FAK_ex:13,15 _and FAK_ex:13,15,16 _were much less abundant than the others, requiring 40 PCR cycles for a good detection. To compare directly the pattern of inclusion of exons 13, 14 and 16 during development, we evaluated the total amount of PCR products containing each of these exons (Fig. 6D). Two patterns were clearly apparent. On the one hand the inclusion of exon 13 was very weak at E12, and increased gradually during development. On the other hand inclusion of exon 14 and 16 increased rapidly between E12 and E20 and remained stable or decreased slightly afterwards.

Since amplification from brain tissue does not allow to distinguish between cell types, we examined the exonic pattern of FAK in cultured neurons. Only 5 FAK variants were detected in E16 cortical neurons (Fig. 6C right lane). The most abundant variants were FAK_ex:15,16_, FAK_ex:14,15,16 _and FAK_ex:13,14,15,16 _while the detection of FAK_ex:13,15,16_, required 40 PCR cycles and was only detected as traces. These results demonstrate that alternative splicing occurs in neurons and that most variants expressed in these cells contain exon 16. Furthermore FAK_ex:15 _(Fig. 6C, M-F3/M-R2, right lane), which is detected in whole brain RNA is only expressed as traces in neurons, suggesting that it probably originates mostly from non-neuronal cells in whole tissue.

Altogether, these results show that the inclusion of the three alternative exons is already taking place at E12. It increases dramatically between E12 and E15, in the case of exons 14 and 16 coincident with the bulk of neuronal differentiation, whereas the increase is more prolonged in the case of exon 13. This difference suggests that the mechanism regulating the inclusion of these various exons in neurons are independent from each other.

### Alternative splicing of FAK exons 13, 14, and 16 in rat tissues

**Figure 7:**
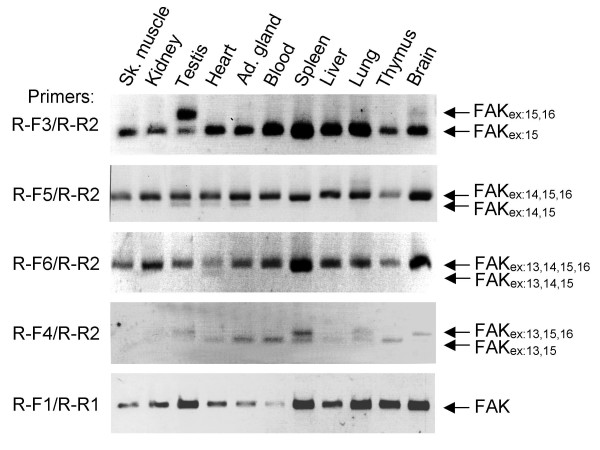
**Alternative splicing of exons 13, 14 and 16 in rat tissues**. Total RNA was extracted from various rat tissues. The expression of alternative splice FAK variants containing the various possible combinations of exons 13, 14 and 16 in various rat tissues was monitored by RT-PCR as described in Fig.6A.

We screened various rat tissues for the expression of FAK transcripts containing exons 13–16 with the RT-PCR approach described above. Using primers flanking the alternative exons (R-F2/R-R2), we detected in most tissues a single PCR product with a migration on agarose gels expected for a fragment devoid of alternative exons (FAK_ex:15_). An additional longer PCR product was detected in skeletal muscle and testis, and two additional fragments in brain (data not shown). Total FAK transcripts were evaluated in each tissue (R-F1/R-R1). To determine the exact combination of alternative exons in each tissue we used exons boundaries-specific primers (R-F3, R-F4, R-F5, R-F6), as described above. Transcripts containing no alternative exon (FAK_ex:15_) were present in all tested tissues (Fig. 7, R-F3/R-R2). Transcripts with alternative exon 13 alone (FAK_ex:13,15_) were detected at low levels in adrenal gland, blood, spleen, and thymus, and at very low levels in heart, liver and lung (Fig. 7, R-F4/R-R2). Transcripts with alternative exon 14 alone (FAK_ex:14,15_) were detected at very low levels in testis, heart and adrenal gland (Fig. 7, R-F5/R-R2). Transcripts with exon 16 alone (FAK_ex:15,16_) were highly expressed in testis and detected in brain (Fig. 7, R-F3/R-R2). FAK_ex:14,15,16 _was detected in all tested tissues (Fig. 7, R-F5/R-R2). Transcripts with associated exons 13 and 14 (FAK_ex:13,14,15_) were detected at very low levels in heart (Fig. 7, R-F6/R-R2). FAK_ex:13,15,16 _was detected in testis, spleen, lung and brain (Fig. 7, R-F4/R-R2) and FAK_ex:13,14,15,16 _was detected in all tissues examined (Fig. 7, R-F6/R-R2). As in mouse brain, the isoforms including exon 13 appeared to be less abundant since they required 35–40 amplifications cycles. These results combined with those obtained in mouse brain show that exons 13, 14 and 16 can be included in combination or independently of each other. Overall three transcripts are more widely and highly expressed than the others: FAK_ex:15_, FAK_ex:14,15,16 _and FAK_ex:13,14,15,16_. Thus, in non-neuronal tissues FAK_ex:15 _is the major isoform, except in testis where FAK_ex:15,16 _is predominant, as previously reported using different approaches [40,41]. These results indicate that the inclusion of exons 13, 14, and 16 is tightly regulated in a tissue-dependent fashion.

### Alternative splicing of FAK exons 13, 14, and 16 in human brain

**Figure 8:**
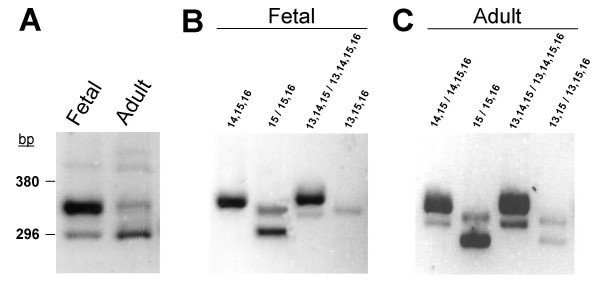
**Alternative splicing of FAK exons 13, 14 and 16 in human brain**. (**A**). Analysis of FAK variants by RT-PCR with flanking primers. The position of molecular markers 296 bp and 380 bp correspond to the size of the expected PCR products for FAK_ex:15_, and FAK_ex:13,15 _respectively. The expression of alternatively spliced FAK transcript was detected in human fetal (**B**) and adult brain (**C**) by RT-PCR as described in Fig. 6A. The exons included in each PCR products reflecting the expression of the different FAK variants are indicated.

Although alternatively spliced variants of FAK exist in humans (see above), no information is available concerning their expression in brain. To search for FAK splice variants containing exons 13, 14 or 16 in human brain we used the same RT-PCR approach as described above (Fig. 6A), with primers matching the human FAK nucleotide sequence. Using primers (H-F2/H-R2) flanking exons 13, 14 and 16, we detected multiple FAK transcripts in fetal and adult human brain RNA (Fig. 8A). In fetal brain, the predominant PCR product corresponded to the expected size of FAK_ex:14,15,16 _while a band of the size of FAK_ex:15 _was also present (Fig. 8A). In adult brain FAK_ex:15 _was the most abundant variant (Fig. 8A). In both types of samples, longer PCR products presumably including exons 13 and 14 or 16 were barely detectable (Fig. 8A).

We then performed RT-PCR with primers H-F3, H-F4, H-F5, H-F6, selective of the different combinations of exons 13,14 and 16, and the reverse primer H-R2 (see Fig. 6A) to determine precisely the expression of each FAK transcript. In fetal human brain RNA the main transcripts amplified were FAK_ex:15_, FAK_ex:14,15,16 _and FAK_ex:13,14,15,16 _(Fig. 8B). FAK_ex:15,16 _and, to a lesser degree, FAK_ex:13,14,15 _and FAK_ex:13,15,16 _were also detected (Fig. 8B). Interestingly, in adult brain, although the general pattern was the same as in fetal brain, all exons combinations were amplified, including FAK_ex:14,15 _and FAK_ex:13,15 _(Fig. 8C). These results demonstrate that alternative splicing of FAK exons takes place in human brain, as in mouse or rat. In human fetal brain samples, results were very similar to those in fetal mouse brain at E15-20. In contrast, two noticeable differences were observed in human adult brain. First, the levels of expression of FAK_ex:15 _appeared higher than those of FAK_ex:14,15,16_. This may be due to the fact that FAK_ex:15 _is expressed in non-neuronal cells, including glial cells, which represented a higher proportion in adult human tissue samples. Second, all combinations of exons were detected, even if most of them were found at very low levels. This may indicate a larger variety of splicing mechanisms and/or cell types included in the samples.

## Discussion

The present study provides a global and updated view of the FAK family of non-receptor tyrosine kinases. FAK and PYK2 share a high degree of amino acid sequence identity (~45%) and nearly identical intron-exon structure (data not shown). In contrast to a previous report [33], the phylogenetic analysis presented here suggests that PYK2 and FAK share a common ancestor posterior to the appearance of the echinoderm branch. This different result is probably due to the inclusion of a larger number of FAK sequences in the present study, including cnidarian, arthropod and urochordate sequences. The proposed duplication event leading to the emergence of two FAK family members is in agreement with chordate genome evolution in which one or several duplications occurred after the separation of craniates and cephalochordates, before the emergence of teleosts [64,65]. The duplication in the early stage of vertebrate evolution was also proposed for other tyrosine kinases families including platelet-derived growth factor receptor (PDGFR) and Src tyrosine kinase families [66]. In addition, two FAK sequences were found experimentally in *Danio rerio *[35] and predicted in *Takifugu rubripes *(Ensembl V38) in agreement with the additional genome duplication in teleost ancestors [45]. Similarly, two PYK2 genes are predicted in *Danio rerio *(Ensembl V38), although only one has yet been annotated in *Takifugu rubripes *(Ensembl V38). Thus, FAK and PYK2 can be considered as paralogous genes in vertebrates. Vertebrate FAK sequence is closer than PYK2 to FAK in urochordate, echinoderma and other non vertebrate species, suggesting that PYK2 has undergone a more rapid evolution, presumably linked to its novel functions.

The highest degree of conservation in FAK family proteins is found in the FERM, kinase and FAT domains, whereas the linker regions are less conserved. Interestingly, the autophosphorylated tyrosine and the surrounding conserved motif, in the linker between FERM and kinase domains, is absent from *C. elegans *genome sequence, suggesting that it is not essential to FAK function. The kinase-FAT linker region was also poorly conserved with multiple insertions/deletions in various species. Not surprisingly it is in these linker regions that conserved alternative splicing events take place in vertebrates, including around the autophosphorylated tyrosine (exons 13, 14, 16) in FAK and upstream from the FAT domain (exon 31) in FAK and the short isoform of PYK2 [38,67].

We updated the annotation of murine and human genes to take into account the conserved exons, based on the analysis of transcripts and EST databases as well as RT-PCR experiments. Murine and human FAK genes span 140 and 230 Kb respectively (see additional figure 2). The murine and human FAK genes include at least 40 and 44 exons, respectively. Four 5'-non coding exons (-4 to -1) and 34 coding exons (1 to 34) are conserved in both species (81–100% identity). We also show that the promoter regions of full length FAK and FRNK, previously identified in human and mouse, respectively [37,47] are conserved between the two species, allowing to identify novel conserved regions of putative regulatory interest. Interestingly, the existence of two alternate gene products, full length PYK2 and PRNK, has been reported in the case of PYK2 [38], suggesting that the two promoters are an ancient and conserved feature of this tyrosine kinase family.

Most of the translational control occurs at the level of initiation, implicating the 5'-untranslated (5' UTR) region as a major site of translational regulation [49]. FAK mRNAs cloned from rat brain contained various 5' UTR sequences suggesting a tight translational regulation [39]. The present work expands these results since we identified 6 murine and 8 human 5' UTR exons. Four of these exons (-1, -2, -3 and -4) are highly conserved in both species (62–87 % identity), while the remaining exons are not. Cis-regulatory splicing sequences are enriched in exons -4, -2bH and in the intron between exons -2 and -2aH (intron -2aH). We did not find any correlation between the organization of the 5' UTR and the inclusion and exclusion of specific alternatively spliced coding exons in FAK transcripts, although a more systematic study would be necessary to draw definite conclusions.

Within the coding sequence the present study shows that the previously described exons 13, 14, 16, and 31 are highly conserved in vertebrates. A number of FAK transcripts containing additional sequences have been reported, especially in human. We examined in detail these variants to determine whether they could encode for novel forms of FAK with interesting biological properties. One variant transcript [GenBank: BC028733] would encode a FAK isoform deleted of the N-terminal of FAK but including Tyr-397 and the kinase domain. We also characterized FAK transcripts containing modifications that would result in various C-terminal truncations. The presence of either exon 18a or of an extended exon 21 would encode isoforms of FAK deleted of their C-terminal moiety and most of the kinase domain. The inclusion of exon 23a would result in FAK isoforms containing an intact kinase domain but deleted of the C-terminal proline-rich regions and the FAT domain. We also found transcripts lacking exon 28 (no change in ORF) or exon 29 (deletion of FAT domain). Another FAK transcript in which exon 26 (present nomenclature) is spliced out has been reported. This transcript is particularly interesting since exon 26 of FAK corresponds to the exon 23 of PYK2 which is also deleted in an alternative splice variant of PYK2 [38,67]. These exons encode residues localized in the C-terminal region, between the proline-rich motifs PR2 and PR3, suggesting that their deletion could have similar functional consequences. Although we did not detect a significant expression of this FAK transcript in human brain, it could be expressed in other tissues similarly to the PYK2 alternative splice variant [67].

The presence of several of these variant transcripts in human brain was supported by our RT-PCR experiments, which showed that they are expressed at low levels, similar to FAK transcripts containing exon 13. However, exons 18a and 23a are not conserved in mouse, and non-conserved alternative splicing events are less likely to generate functional proteins than those which are conserved [68]. Thus, it remains to be established if these species-specific transcripts are translated into proteins in human tissues. Transcripts containing exon 18a may be degraded since inclusion of this exon results in a change in ORF and a stop codon in exon 19 following the nonsense-mediated decay (NMD) rules [52]. NMD is an mRNA surveillance pathway responsible for the degradation of abnormal mRNAs containing premature translation termination codons (PTCs) which encode truncated proteins potentially harmful for the cell [69]. Altogether the lack of evolutionary conservation of these alternative FAK transcripts does not support an important biological role. As a matter of fact, several of these non-conserved alternatively spliced FAK exons discussed here exhibit a strong homology with repeated elements. The 5'-untranslated exons -2aM in mouse and the human -3aH and -3bH are likely to derive from Alu elements. The primate specific genomic insertion of exons 18a and 23a could be the consequence of LINE insertion. The exonization of intronic transposable elements (ALU/LINE) is a well characterized phenomenon in mouse and human and could explain the diversity and the species specificity of those FAK gene exons [54].

Since our results highlighted a complex regulation of FAK mRNA alternative splicing we searched in exons 13, 14, 16 and 31 and in their flanking introns for cis-regulatory sequences, conserved in mouse and human, known to influence alternative splice site selection [70]. Exons 13 and 14 were recognized as alternative conserved exons according to Yeo et al [56]. We found an overrepresentation of exon splicing silencers (ESS) or enhancers (ESE) in exons 14 and 16 respectively. We also identified, in the intronic region between exons 13 and 14, a high frequence of a well characterized intronic splicing enhancer sequence (UGCAUG) regulating tissue-specific alternative exons inclusion/exclusion [63,71]. We determined that this hexanucleotide is conserved in human, mouse, rat, dog and chicken FAK (data not shown) suggesting that it could be involved in the regulation of alternative splicing of exon 13 and/or 14. Functional evidences have shown that UGCAUG binds various tissue-specific isoforms of mammalian Fox-1 splicing factors with a high specificity [72,73]. Fox-1 family factors can act as splicing enhancers or repressors depending on the tissue, the isoform, as well as the localization of the hexanucleotide binding site (upstream or downstream) and its distance from the targeted alternative exon. The effect of Fox isoforms on the alternative splicing of exons 13 and 14 remains to be established and predictions are difficult since both exons can be alternatively spliced. However, in muscle cells Fox-1 was shown to act as a splicing repressor of exons normally skipped in muscle but used in other tissues [73] and this effect required the localization of the hexanucleotide upstream from the targeted exon which would correspond here to exon 14. This type of regulation could be responsible for the absence of exon 14 in FAK transcripts in some tissues. On the other hand, the UGCAUG sequences reported to activate splicing are located downstream from the regulated exon [63] concerning here the exon 13 of FAK. Furthermore, the UGCAUG sequence and various Fox isoforms have been shown to activate the splicing of neuron-specific alternative exons [63,71] such as the N30 exon of the non-muscle myosin heavy chain II-B [74] or the N1 exon of Src [73]. Thus, the presence of multiple cis-exonic and intronic sequences regulating positively or negatively alternative splicing can be correlated with the complex RT-PCR expression profile of FAK isoforms containing various combinations of exons 13, 14 and 16 that we observed in adult tissues and during development.

The alternative exons encoding boxes 28, 6, 7 and PWR (exons 13, 14, 16 and 31) are highly conserved in vertebrates supporting an important biological role. Here, we demonstrate by RT-PCR that, as expected, alternative splicing of exons 13, 14 and 16 takes place in human. The study of the pattern of inclusion of exons 13, 14 and 16 suggests that they are independent from each other and subjected to tissue and development-specific regulatory mechanisms. For instance, FAK_ex:15_(FAK^0^), FAK_ex:14,15,16 _(FAK^6,7^) and FAK_ex:13,14,15,16 _(FAK^6,7,28^) can be detected at various levels in all tissues tested while the expression of other exons combinations is restricted to very few tissues, usually at very low levels (e.g. FAK_ex:14,15 _(FAK^6^) in testis, heart, and adrenal gland, and FAK_ex:13,14,15 _(FAK^28,6^) in heart). Overall, FAK_ex:15 _(FAK^0^) is the most abundant transcript, except in testis and brain in which FAK_ex:15,16 _(FAK^7^) and FAK_ex:14,15,16 _(FAK^6,7^) respectively, are preponderant.

Our results demonstrate that the majority of transcripts expressed during brain development or in adult brain contain exons 14 and 16 (encoding boxes 6 and 7), in agreement with previous results obtained with different approaches [40]. During mouse brain development the inclusion of these exons increases dramatically between E12 and E15. Interestingly, this period (E12-E15) corresponds to the initial stages of cortical development in the brain, including cortico-pial basement membrane formation and neuronal migration, both processes requiring FAK [22,75]. The preponderance of FAK transcripts containing exons 14 and 16 in human and rodent brain is particularly interesting since the inclusion of the corresponding peptides in FAK isoforms increases significantly the autophosphorylation rate [40,41]. This effect results from the relief of the inhibition by the FERM domain, and from the ability of FAK including boxes 6 and 7 (or box 7 alone) to undergo intramolecular autophosphorylation [41,42]. These specific properties have important consequences since they suggest that the recruitment of these alternatively spliced FAK isoforms may lead to their autophosphorylation without requirement for the clustering of multiple FAK molecules in contrast to what occurs in focal adhesion. Interestingly, the expression of FAK transcripts containing exon 16 is not restricted to brain. FAK_ex:15,16 _is the most abundant FAK transcript in testis in which the autophosphorylation of FAK is essential for specific actin-based adherens junctions assembly and disassembly between Sertoli and germ cells [76]. FAK_ex:14,15,16 _and FAK_ex:13,14,15,16 _are also expressed, although at a much lower level than in brain, in the other tissues tested. The inclusion of box 6 (encoded by exon 14) also increases autophosphorylation and its effect is additive with that of box 7 (exon 16) [41]. In contrast, the function of box 28 in FAK is not known. Our results showing the strong evolutionary conservation of boxes 28 and 6 in vertebrates support an important functionnal role, yet to be established.

## Conclusion

The FAK gene family includes one orthologue in non-vertebrate metazoans and two paralogue genes in vertebrates, FAK and PYK2. In vertebrates FAK is subjected to conserved alternative splicing of several non-coding and coding exons which are likely to have an important biological function. In contrast, other variations due to non-conserved alternative splicing may be the consequence of the presence of transposable elements in the genome and have no function or be possibly involved in regulatory mechanisms. FAK is known to play an important role in human pathology, especially in cancer invasiveness and metastasis [24]. Since alterations in splicing mechanisms are frequent in cancer cells [77,78] it will be important to determine whether such mechanisms could alter FAK properties, either by modulating the mRNA levels, or by changing the coding sequence of expressed proteins.

## Methods

### FAK gene annotation

New exons of FAK gene were identified and annotated from alignment of ESTs or mRNAs with FAK genomic sequences from human [RefSeq:NT_008046] and mouse [RefSeq:NT_039621] using either ClustalW [79,80], Blast2Sequences or BLASTN [81]. Exons 13, 14, 16 and 31 were searched in FAK gene from chimpanzee [Ensembl:ENSPTRG00000020624], dog [Ensembl:ENSCAFG00000001217], chicken [Ensembl:ENSGALG00000016171], frog [Ensembl:ENSXETG00000023764], fugu [Ensembl:NEWSINFRUG00000124163, Ensembl:NEWSINFRUG00000135169], zebrafish [Ensembl:ENSDARG00000004672, Ensembl:ENSDARG00000040197]. Repeated elements were identified with the RepeatMasker program [82]. Alternative splicing regulatory elements were searched using Rescue-ESE [59,83,84], Acescan2 [56,85] and EBI ASD tools [60-62,86].

### Phylogeny

The sequence alignment of full-length FAK and PYK2 proteins was obtained using ClustalW followed by manual adjustments. The resulting alignment was represented using Genedoc [87,88] (see additional file 1). Evolutionnary distant and predicted FAK sequences were used. To avoid poorly aligned positions, conserved blocks distributed across the full length FAK sequences were selected with the Gblocks program [44]. The phylogenetic analysis based on Maximum Likelihood was carried out with Phyml [89-91]. Distance analysis was performed with Protdist to generate distance matrix and the Neighbour Joining algorythm to infer trees [92]. Trees were drawn with Njplot [93]. Phylogenetic studies were also performed using FERM and kinase domains alignment corresponding to residues 33 to 361 and 415 to 677 of the human FAK sequence [Swiss-Prot: Q05397], respectively. The phylogenetic analysis of the FAT domain (residues 914 to 1043) did not fit with known evolutionnary data (branching of nematodes, insects and echinoderma) since this domain is poorly conserved between the different species. The bootstrap support values (100 replicates) indicated above the respective nodes, were computed with Phyml. Additional accession numbers of FAK sequences: mouse [GenBank: AAA37592], rat [Swiss-Prot: >O35346], frog [Swiss-Prot: Q91738], chicken [Swiss-Prot: Q00944], zebrafish [GenBank: AAK31154, GenBank: AAP36454], sea urchin [GenBank: AAN38839.1], fruitfly [GenBank: AAF15292]. Accession numbers for PYK2 sequences: human [RefSeq:NP_775268.1], mouse [RefSeq:NP_766086.1], rat [Swiss-Prot: P70600], zebrafish [RefSeq:NP_997735.1]. Accession numbers for FAK and PYK2 predicted sequences : chimp FAK [Ensembl:ENSPTRP00000035264], fugu FAK1 and FAK2 [Ensembl:NEWSINFRUP00000131164, Ensembl:NEWSINFRUP00000143246] mosquito FAK [Ensembl:ENSANGP00000008377], honeybee FAK [RefSeq:XP_396962.1], *C. elegans *FAK [GenBank: AAK85457.1], hydra [GenBank: AAW21807.1], hydractinia FAK [GenBank: AAV97963], *C. intestinalis *FAK [Ensembl:ENSCINP00000007661], chimp PYK2 [Ensembl:ENSPTRP00000034416], chicken PYK2 [Ensembl:ENSGALP00000026682], frog PYK2 [Swiss-Prot: Q5XH98] fugu PYK2 [Ensembl:NEWSINFRUP00000140223], zebrafish PYK2-2 [Ensembl:ENSDARP00000036466].

### Cell culture

Primary neuronal cultures established from cerebral cortices of embryonic day 16 C57BL/6 mice were performed as previously described [94]. The cells were seeded in plates coated with 25 μg/mL poly-L-lysine (Sigma P-8638) and cultured at 37°C in a humidified atmosphere of 5 % CO_2 _using neurobasal media (Invitrogen cat. 21203-049) supplemented with 2 % B27 (Invitrogen cat.17504-044) and 0.5 mM L-Glutamine. Neurons harvested at J_0 _correspond to cells lysed 4 hours after plating.

### Reverse Transcription-Polymerase Chain Reaction (RT-PCR)

Total RNA was extracted from rat and mice tissues or neuronal cultures with RNA NOW (Biogentex, Inc, cat. BX-102) as recommended by the manufacturer. Total RNA from adult and fetal human brains were purchased from BD Biosciences. One microgram of total RNA was reverse transcribed in 20 μl final volume, using oligo(dT) and the ImProm-II Reverse Transcription System (Promega). PCRs were performed in 25 μl with the Multiplex PCR kit (QIAGEN), including 0.5 to 1.5 μl cDNA (depending of the experiment) and 0.8 μM of the appropriate primers. PCR amplifications consist in an initial denaturation step (95°C for 15 min) followed by 30, 35 or 40 cycles of amplification (94°C, 30 sec; 58°C, 1.30 min and 72°C, 1.30 min) and a final extension of 10 min. PCR products were analyzed on 2–4% agarose gel in 0.5× TAE buffer. Single fragment PCR products were directly purified using the QIAquick PCR purification kit and sequenced. In case of simultaneous amplification of several fragments, each fragment was excised from agarose gel, recovered using the QIAEX II gel extraction kit and sequenced. The cDNAs were first used to amplify GAPDH transcripts before investigating the expression of FAK transcripts.

The total level of FAK transcripts (independently of the inclusion/exclusion of exons 13, 14 and 16) was examined using forward and reverse primers located in exons 7 and 9 respectively. To examine the global pattern of FAK transcripts with distinct combinations of alternative exons 13, 14 and 16 (corresponding to boxes 28, 6 and 7), RT-PCR were performed with primers flanking this region (forward primer in exon 12 and reverse primer at the exons 18–19 boundary). Improved detection of alternative transcripts expressed at low levels can be achieved by using primers spanning each of the expected exonic junction [95]. A set of 4 forward primers were designed including the last 12 nt located at the 3' end of the upstream exon and the first 12 nt of the following exon, (except for H-F5 and R-F5, 25 mers). These 4 forward primers were individually coupled with the unique reverse primer (see above) (Figure 6A). Each pair of primers allows the specific detection of two FAK transcripts, differing by the presence or absence of exon 16. A total of 8 alternative transcripts may be expected (see Figure 6). FAK primers specific for human, rat and mouse species were designed according to the sequence reported for each species (Accession numbers NCBI Gene ID:5747, NCBI Gene ID: 25614, NCBI Gene ID:14083). The expression of other putative alternative human FAK transcripts was also examined (see Table 1): [RefSeq:NM_005607, GenBank: BC028733, GenBank: L05186, GenBank: BC035404]. All primers sequences are reported in additional file 8.

Quantification of PCR products was done by scanning pictures of agarose gels stained with ethidium bromide and measurement of relative optical density (Scion image program, Scion Corporation). The values obtained for FAK variants were normalized to GAPDH and expressed as percentage of the maximal value.

## Authors' contributions

JMC conducted the various data base searches and sequence analyses, had a critical participation in the design of the studies and the analysis of data, and drafted the manuscript. ER conceived, designed and performed all the RT-PCR experiments, and revised the manuscript. JAG and HE conceived, designed and coordinated the study, and finalized the manuscript. All authors read and approved the final manuscript.

## Supplementary Material

Additional File 1 **Alignment of FAK family proteins**. Dashes denote gaps. Residues conserved in all species are shaded in dark. Residues conserved in at least 80 or 60% of the species examined are shaded in green and grey, respectively. Numbers on the right indicate the position in the whole sequence of the last alphabetic sequence character in the sequence line. Alignment gaps are indicated by (-). The localizations of the FERM, kinase and FAT domains on FAK human sequence are indicated in the Methods section. The sequence alignment was obtained using ClustalW (see Methods) and optimized manually. Click here for file

Additional File 2 **Complete genomic organization of the human and mouse FAK genes**. Intron sizes are shown to scale. Promoters (Prom) are represented as dots. Start codons are indicated (FAK-ATG, FRNK-ATG). Alternatively spliced exons are boxed. Exons 18A and 23A are specific of primate genomes. Click here for file

Additional File 3 **Pairwise conservation profile of human and mouse FAK promoters and identification of transcription factor binding sites**. Alignment of the 5' sequence of the mouse FAK gene with the human FAK promoter [GenBank: AY323812]. The multiple-sequence local alignment tool Mulan [96] was used to obtain a pairwise conservation profile of the human and putative mouse promoter. Dashed blue line indicates the minimal human FAK promoter, and the +1 nucleotide corresponds to the human transcription initiation site located approximatively 110 Kb from the start codon of the human FAK gene [47] (see Fig. 3). Short dark lines indicate the length and similarity (mouse versus human) of individual blocks of nucleotides across the identified conserved region. Two evolutionary conserved regions, ECR1 and ECR2 (underlined with brown lines), longer than 100 bp and sharing more than 70% similarity overall were identified. Red histograms indicate the percentage of similarity of individual blocks of nucleotide within ECR1 and ECR2. The MultiTF tool (threshold = 0.95) was used to predict conserved transcription factor binding sites (TFBS) between the mouse and human promoters. The identified TFBS (rectangles) are mainly localized in ECR1 and ECR2. Most TFBS previously predicted in the human promoter [47] were found to be conserved in mouse (closed rectangles). Novel putative conserved TFBS identified in this study are indicated with open rectangles. Click here for file

Additional File 4 **Stacked-pairwise conservations profile of human, mouse and chicken FRNK promoters and identification of transcription factor binding sites**. The promoter of chicken and mouse FRNK has been localized in the intron between exon 22 and exon 23. To identify the human FRNK promoter we aligned independently the sequences of this intron in both species with the corresponding human intron using the Mulan program. The conservation profile shown here focuses on the intronic 7 Kb sequence 5' to exon 23 since no conserved region were observed upstream in the intron. The +1 nucleotide corresponds to the mouse transcription initiation site previously reported [48] and located approximatively 1 Kb from the translation start codon of FRNK. Three evolutionary conserved regions, ECR1, ECR2 and ECR3 (underlined with brown lines), longer than 100 bp and sharing more than 70% similarity overall were identified. ECR1 and ECR2 correspond to the previously reported 5'-leader exon of FRNK in chicken [37] and to an enhancer region of the mouse FRNK promoter [48] respectively. We identified a new conserved sequence, ECR3, which could contain regulatory information for FRNK expression. The MultiTF tool (threshold = 0.95) was used to predict conserved transcription factor binding sites (TFBS). Closed rectangles and open rectangles denotes previously reported and novel putative TFBS, respectively [48]. Click here for file

Additional File 5 **Exonic structure of human FAK**. Intron-exon boundaries are depicted by arrows in the nucleic acid sequence. Exon numbers are boxed. Nucleotide and amino acid sequences of alternatively spliced exons are italicized. Click here for file

Additional File 6 **Introns and exons of the mouse and human FAK genes: length and similarity**. Data are based on the analysis of mouse and human sequences of Ptk2 gene (NCBI Gene ID:5747). Click here for file

Additional File 7 **Expression patterns of FAK transcripts containing various combinations of exons 13, 14 and 16 during development**. Three distincts general patterns of expression of FAK alternative transcripts were observed after quantification (see Methods) and normalization of the PCR products reflecting the expression of each transcript. (**A**) FAK_ex:15_, FAK_ex:13,15_. (**B**) FAK_ex:15,16_, FAK_ex:13,15,16_, FAK_ex:14,15,16_. (**C**) FAK_ex:13,14,15_, FAK_ex:13,14,15,16_. Click here for file

Additional File 8 Primers positions and sequences. Click here for file

## Figures and Tables

**Table 1:** Human specific alternative FAK transcripts ^(1)^

**mRNA sequence reference**	**mRNA feature**		**Other sequence containing this modification**	**Found by RT-PCR in brain (2)**	**Predicted amino acid modification**	
				
	**Deletion**	**Insertion/replacement**			**Deletion (3)**	**insertion/replacement**
NM_005607 (stomach)	-	exon -3bH: 77 nt upstream from exon 1	-	No	-	MISADCNLCLPEYDRYLASSKI (alternative ATG in exon -3bH)
-	exon 2	-	human : CB990597 (placenta), CF138217 (lymph), BU838559 (DRG)	Yes	66–121 (exon 2) and 123–1096	STOP codon in exon 3
BC028733 (hippocampus)	exon 1–14	135 nt upstream from exon 15	human : BC043202 (hypothalamus) macaque : CJ459742, CJ459819, CJ460273 (medulla oblongata), CJ445344 (brain)	Yes	1–392	MKYQEVRCLTSFNISVSFPA (Alternative ATG in intron 14)
L05186 and see ref 43	-	exon 18a: 85 nt between exons 18–19	-	Yes	519–1096	ACHYTSLHWNWCRYISDPNVDAAQTPGMQSNNASV*. change in ORF, (*) STOP codon in exon 19
	exon 22–34	15 nt downstream from exon 21	-	Yes	579–1096	GKKSE* (*) STOP codon in intron 21
	exon 24–34	exon 23a : 91 nt between exons 23–24	-	Yes	677–1096	FQNPAQMLPASGRLPNQPCPERENYSFATF* (*) STOP codon in exon 23a
	exon 28	-	human : BQ428019 (melanotic melanoma) dog :XM_851079.1	Yes	834–854	no change in ORF
BC035404 (placenta)	exon 26	-	-	No	744–789	no change in ORF
-	exon 29	-	-	Yes	896–908 (exon 29) and 972–1096	change in ORF in exon 30 and STOP codon in exon 32

(1) See also Fig. 2 or 3.

(2) Experimental results obtained in the present study (see Results for details).

(3) Present nomenclature, see additional file 5
