# Supplementary material for: Organization and post-transcriptional processing of focal adhesion kinase gene
Source: BMC Genomics. 2006 Aug 4;7:198. doi: 10.1186/1471-2164-7-198 (PMC1570463; doi:10.1186/1471-2164-7-198)
Supplement: Additional File 2 — Complete genomic organization of the human and mouse FAK genes. Intron sizes are shown to scale. Promoters (Prom) are represented as dots. Start codons are indicated (FAK-ATG, FRNK-ATG). Alternatively spliced exons are boxed. Exons 18A and 23A are specific of primate genomes. [file 1471-2164-7-198-S2.ppt]

## Slide 1
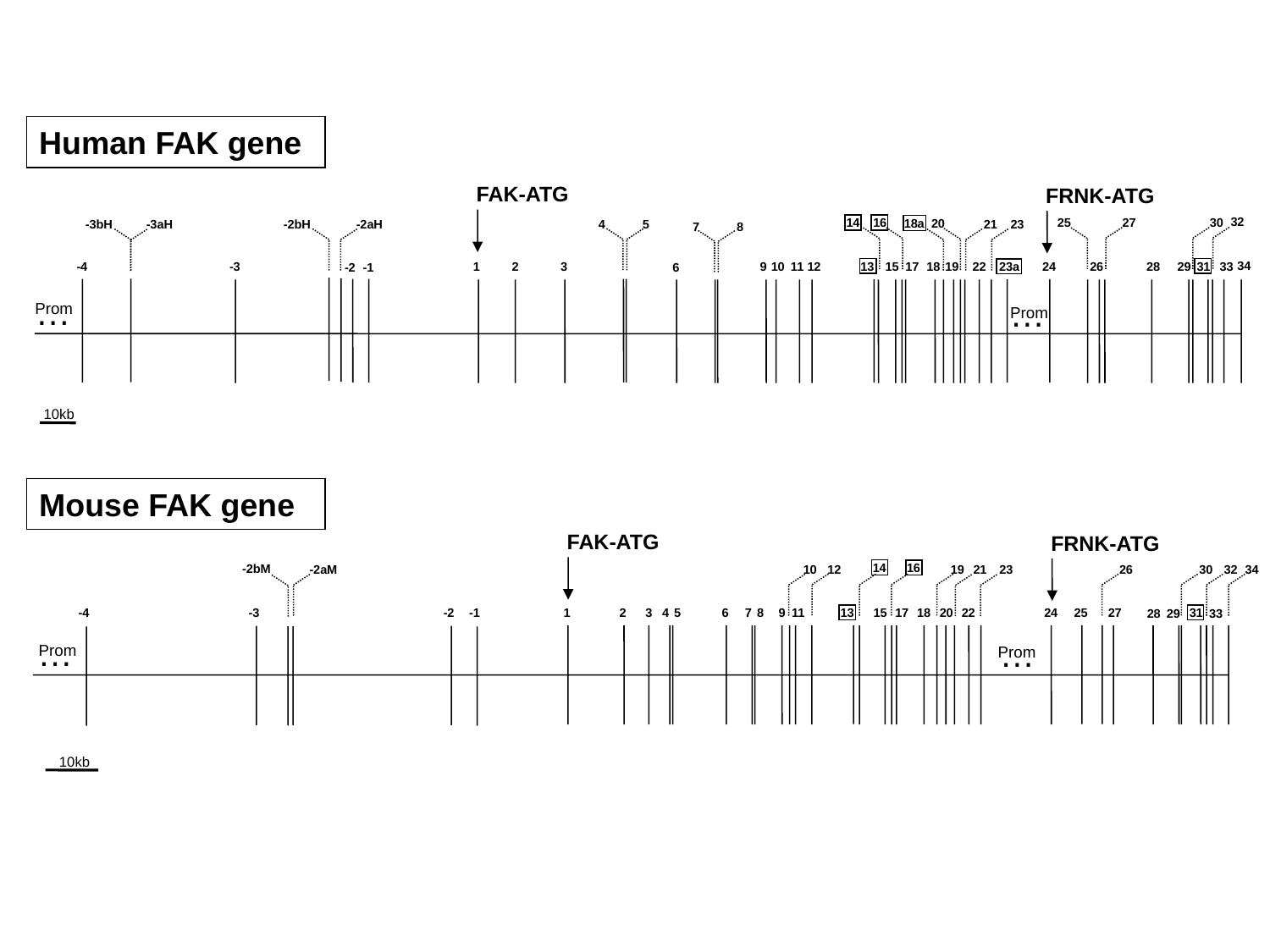

Human FAK gene
FAK-ATG
FRNK-ATG
32
27
30
25
16
14
18a
20
-2bH
-2aH
-3bH
-3aH
5
4
21
23
7
8
34
-4
1
22
29
31
33
-3
2
3
9
12
13
17
18
19
23a
24
26
28
10
11
15
-2
-1
6
…
…
Prom
Prom
10kb
Mouse FAK gene
FAK-ATG
FRNK-ATG
14
16
-2bM
10
12
19
26
-2aM
21
23
30
32
34
7
8
13
15
17
-4
-3
-2
-1
1
2
4
6
9
11
18
20
24
25
27
31
3
5
22
29
33
28
…
…
Prom
Prom
10kb
