# Supplementary material for: Organization and post-transcriptional processing of focal adhesion kinase gene
Source: BMC Genomics. 2006 Aug 4;7:198. doi: 10.1186/1471-2164-7-198 (PMC1570463; doi:10.1186/1471-2164-7-198)
Supplement: Additional File 3 — Pairwise conservation profile of human and mouse FAK promoters and identification of transcription factor binding sites. Alignment of the 5' sequence of the mouse FAK gene with the human FAK promoter [GenBank: AY323812]. The multiple-sequence local alignment tool Mulan [96] was used to obtain a pairwise conservation profile of the human and putative mouse promoter. Dashed blue line indicates the minimal human FAK promoter, and the +1 nucleotide corresponds to the human transcription initiation site located approximatively 110 Kb from the start codon of the human FAK gene [47] (see Fig. 3). Short dark lines indicate the length and similarity (mouse versus human) of individual blocks of nucleotides across the identified conserved region. Two evolutionary conserved regions, ECR1 and ECR2 (underlined with brown lines), longer than 100 bp and sharing more than 70% similarity overall were identified. Red histograms indicate the percentage of similarity of individual blocks of nucleotide within ECR1 and ECR2. The MultiTF tool (threshold = 0.95) was used to predict conserved transcription factor binding sites (TFBS) between the mouse and human promoters. The identified TFBS (rectangles) are mainly localized in ECR1 and ECR2. Most TFBS previously predicted in the human promoter [47] were found to be conserved in mouse (closed rectangles). Novel putative conserved TFBS identified in this study are indicated with open rectangles. [file 1471-2164-7-198-S3.ppt]

## Slide 1
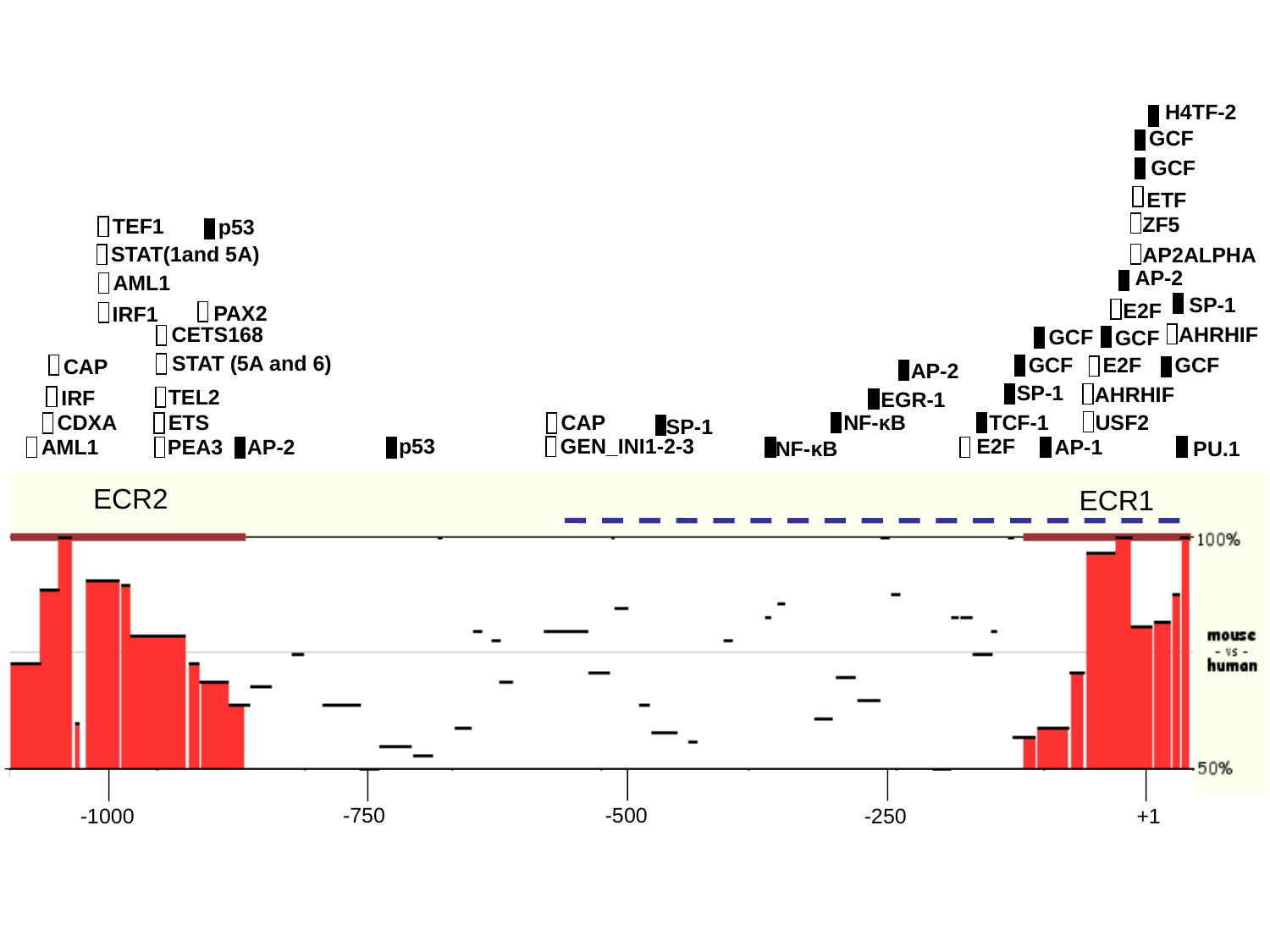

H4TF-2
GCF
GCF
ETF
ZF5
TEF1
p53
STAT(1and 5A)
AP2ALPHA
AP-2
AML1
SP-1
E2F
PAX2
IRF1
CETS168
AHRHIF
GCF
GCF
STAT (5A and 6)
GCF
E2F
GCF
CAP
AP-2
SP-1
AHRHIF
TEL2
IRF
EGR-1
CDXA
ETS
CAP
NF-κB
TCF-1
USF2
SP-1
p53
GEN_INI1-2-3
E2F
AML1
PEA3
AP-1
AP-2
NF-κB
PU.1
ECR2
ECR1
-750
-500
-250
+1
-1000
