# Supplementary figures and images for: Organization and post-transcriptional processing of focal adhesion kinase gene
Source: BMC Genomics. 2006 Aug 4;7:198. doi: 10.1186/1471-2164-7-198 (PMC1570463; doi:10.1186/1471-2164-7-198)

## Slide 1
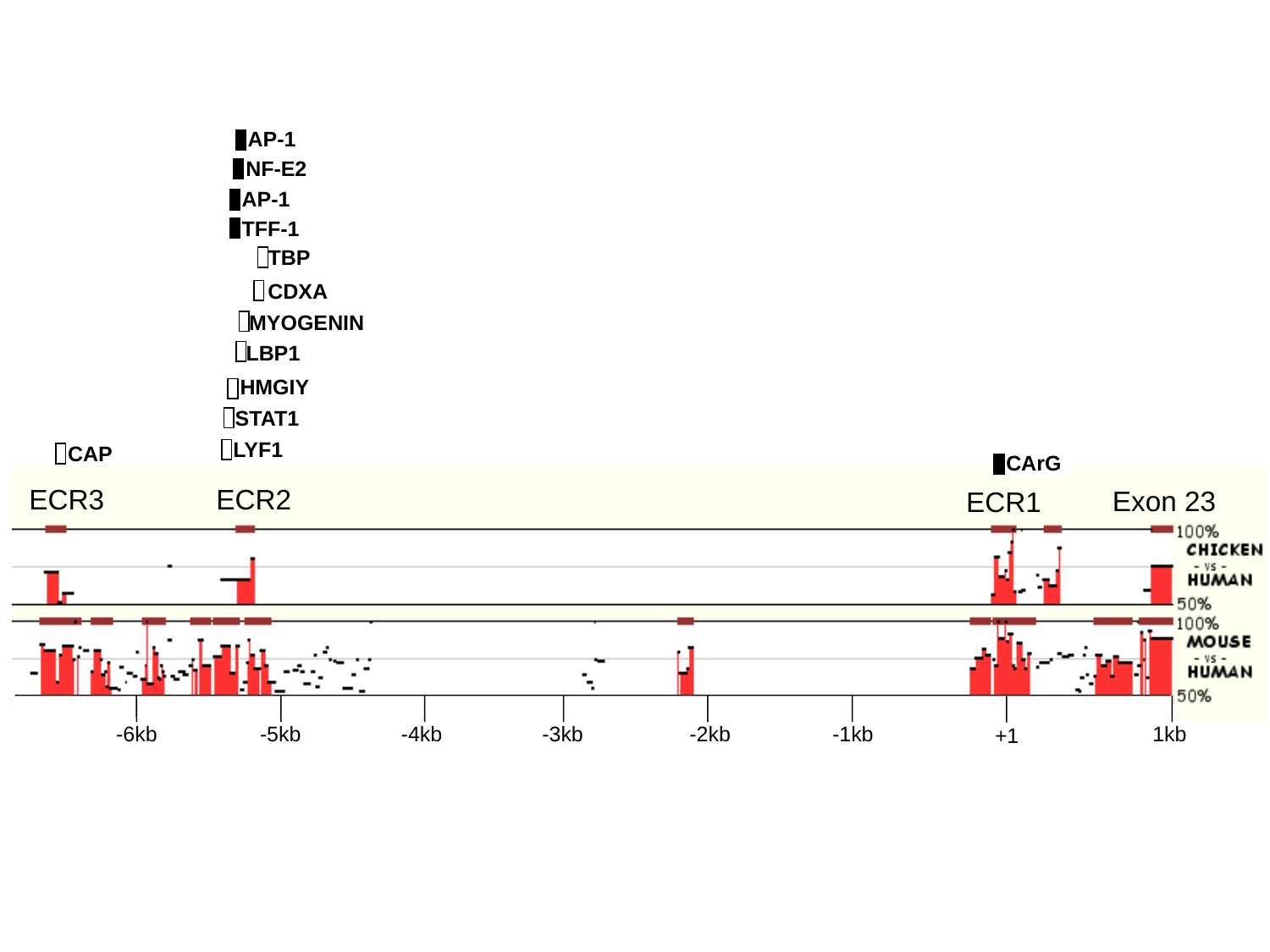

AP-1
NF-E2
AP-1
TFF-1
TBP
CDXA
MYOGENIN
LBP1
HMGIY
STAT1
LYF1
CAP
CArG
ECR3
ECR2
Exon 23
ECR1
-5kb
-4kb
-3kb
-2kb
-1kb
-6kb
1kb
+1

Supplement: Additional File 4 — Stacked-pairwise conservations profile of human, mouse and chicken FRNK promoters and identification of transcription factor binding sites. The promoter of chicken and mouse FRNK has been localized in the intron between exon 22 and exon 23. To identify the human FRNK promoter we aligned independently the sequences of this intron in both species with the corresponding human intron using the Mulan program. The conservation profile shown here focuses on the intronic 7 Kb sequence 5' to exon 23 since no conserved region were observed upstream in the intron. The +1 nucleotide corresponds to the mouse transcription initiation site previously reported [48] and located approximatively 1 Kb from the translation start codon of FRNK. Three evolutionary conserved regions, ECR1, ECR2 and ECR3 (underlined with brown lines), longer than 100 bp and sharing more than 70% similarity overall were identified. ECR1 and ECR2 correspond to the previously reported 5'-leader exon of FRNK in chicken [37] and to an enhancer region of the mouse FRNK promoter [48] respectively. We identified a new conserved sequence, ECR3, which could contain regulatory information for FRNK expression. The MultiTF tool (threshold = 0.95) was used to predict conserved transcription factor binding sites (TFBS). Closed rectangles and open rectangles denotes previously reported and novel putative TFBS, respectively [48]. [file 1471-2164-7-198-S4.ppt]

## Slide 1
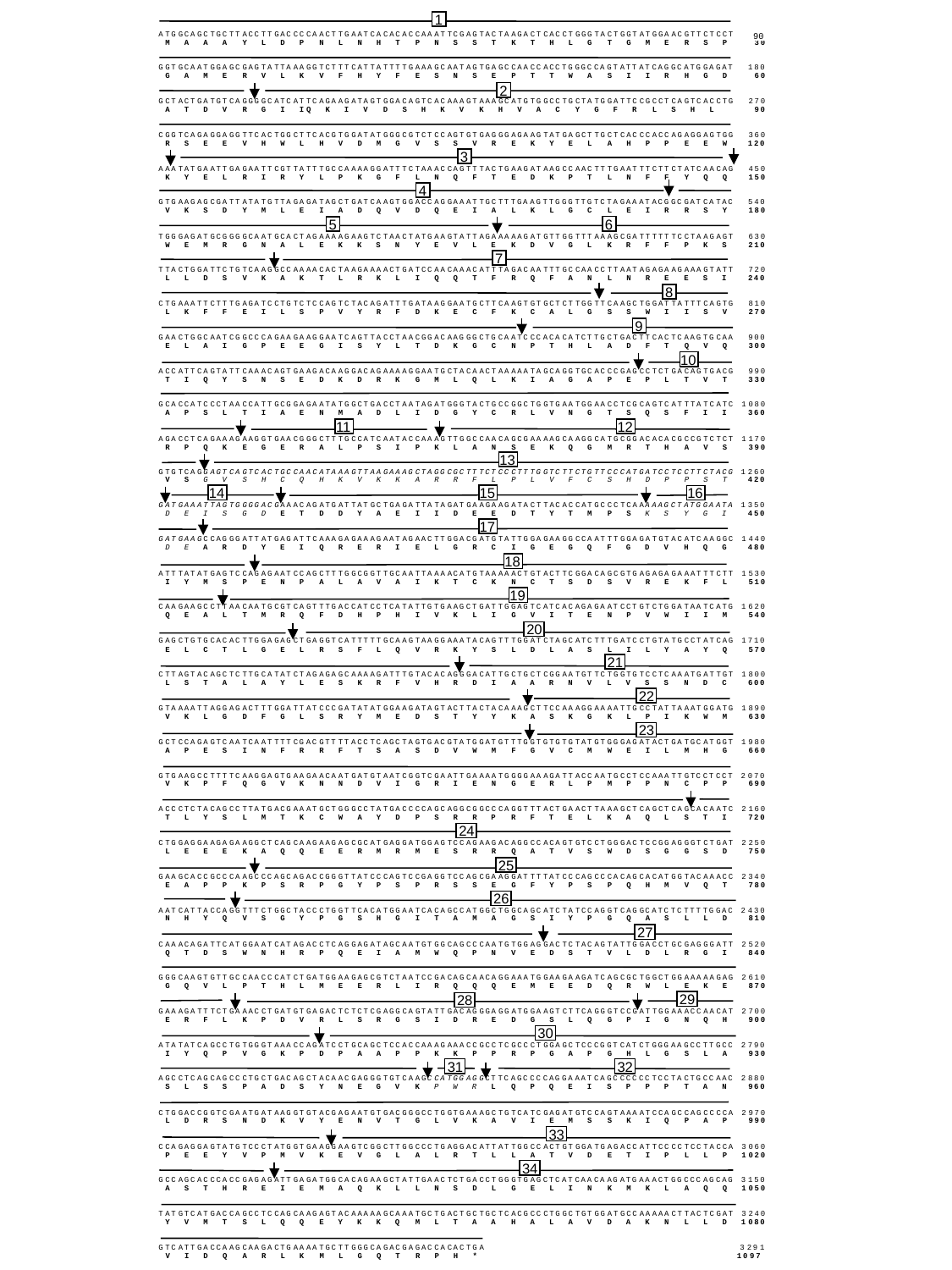

1
90
2
3
4
6
5
7
8
9
10
11
12
13
14
15
16
17
18
19
20
21
22
23
24
25
26
27
29
28
30
31
32
33
34

Supplement: Additional File 5 — Exonic structure of human FAK. Intron-exon boundaries are depicted by arrows in the nucleic acid sequence. Exon numbers are boxed. Nucleotide and amino acid sequences of alternatively spliced exons are italicized. [file 1471-2164-7-198-S5.ppt]

## Slide 1
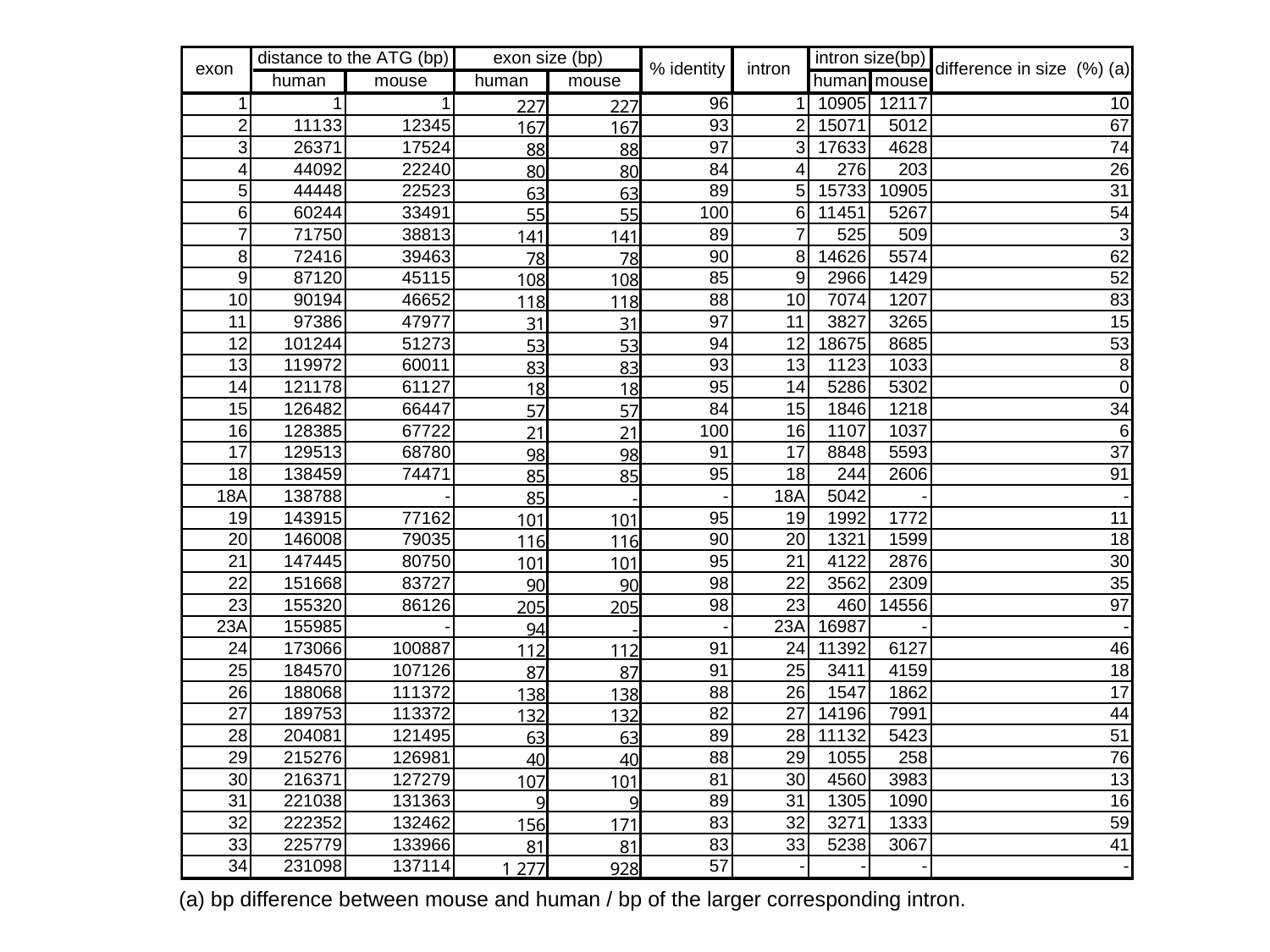

(a) bp difference between mouse and human / bp of the larger corresponding intron.

Supplement: Additional File 6 — Introns and exons of the mouse and human FAK genes: length and similarity. Data are based on the analysis of mouse and human sequences of Ptk2 gene (NCBI Gene ID:5747). [file 1471-2164-7-198-S6.ppt]

## Slide 1
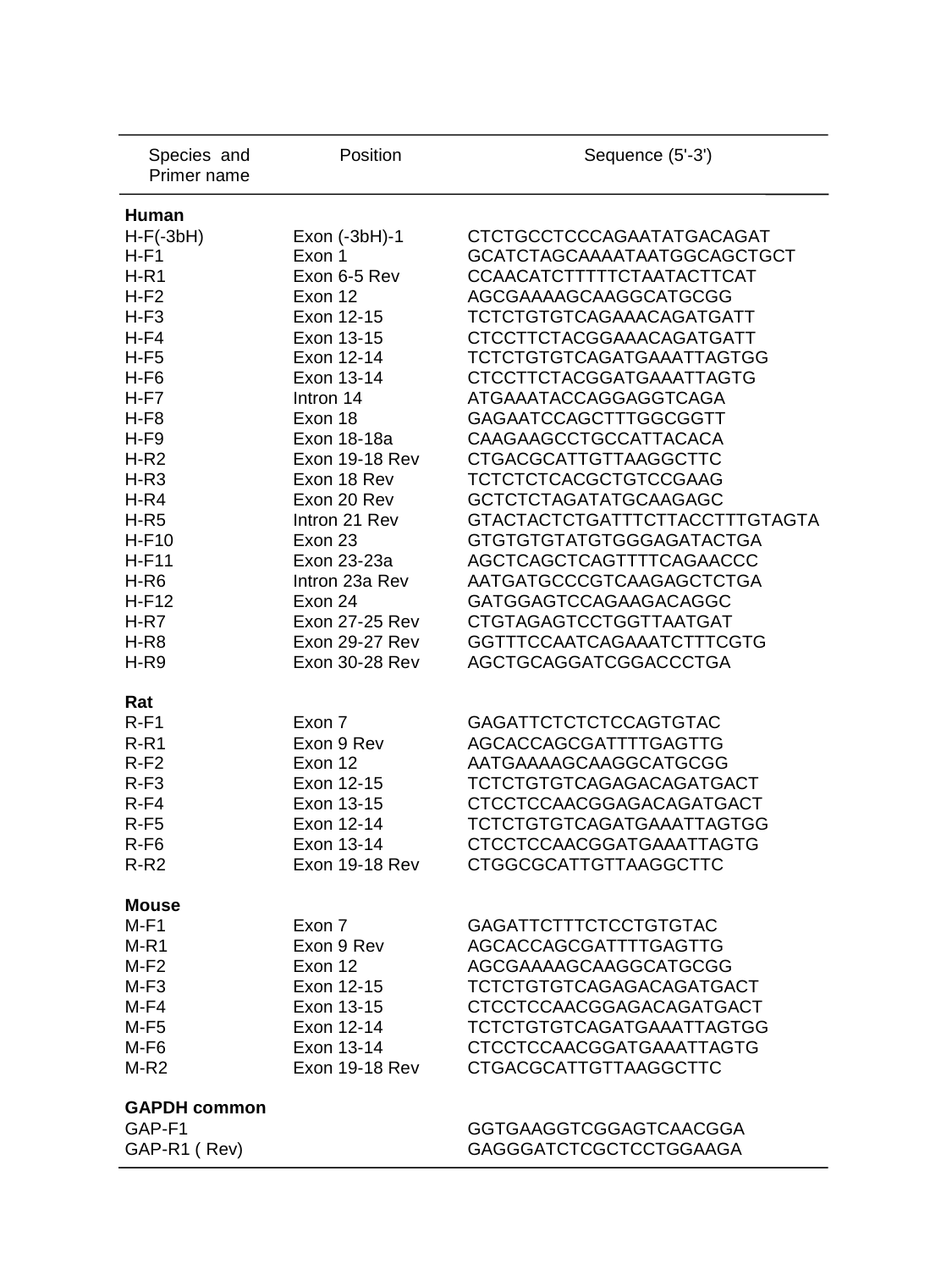

Supplement: Additional File 8 — Primers positions and sequences. [file 1471-2164-7-198-S8.ppt]
