# Supplementary material for: Organization and post-transcriptional processing of focal adhesion kinase gene
Source: BMC Genomics. 2006 Aug 4;7:198. doi: 10.1186/1471-2164-7-198 (PMC1570463; doi:10.1186/1471-2164-7-198)
Supplement: Additional File 7 — Expression patterns of FAK transcripts containing various combinations of exons 13, 14 and 16 during development. Three distincts general patterns of expression of FAK alternative transcripts were observed after quantification (see Methods) and normalization of the PCR products reflecting the expression of each transcript. (A) FAKex:15, FAKex:13,15. (B) FAKex:15,16, FAKex:13,15,16, FAKex:14,15,16. (C) FAKex:13,14,15, FAKex:13,14,15,16. [file 1471-2164-7-198-S7.ppt]

## Slide 1
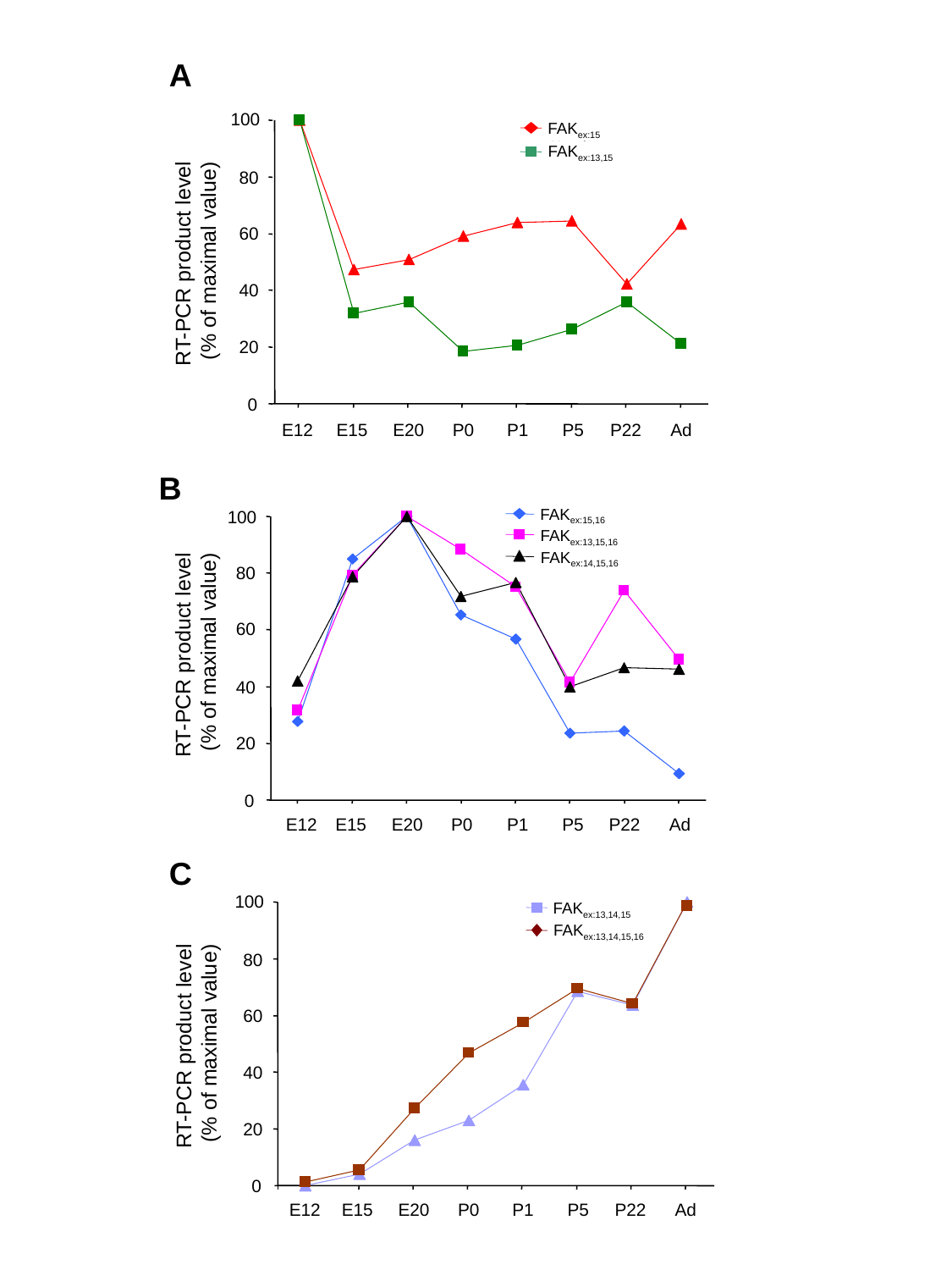

A
100
FAKex:15
O-ctrl
28 -ctrl
FAKex:13,15
RT-PCR product level
(% of maximal value)
80
60
40
20
0
E12
E15
E20
P0
P1
P5
P22
Ad
B
FAKex:15,16
FAKex:13,15,16
FAKex:14,15,16
100
80
60
40
20
0
RT-PCR product level
(% of maximal value)
E12
E15
E20
P0
P1
P5
P22
Ad
C
100
FAKex:13,14,15
FAKex:13,14,15,16
RT-PCR product level
(% of maximal value)
80
60
40
20
0
E12
E15
E20
P0
P1
P5
P22
Ad
